# Predictive Value of Machine Learning in Knee Osteoarthritis Progression: Systematic Review and Meta-Analysis

**DOI:** 10.2196/80430

**Published:** 2025-12-30

**Authors:** Yanwen Liu, Guangzhi Xiao, Youqun Zhang, Xinyi Wang, Junfeng Jia, Aiguo Xie, Zhaohui Zheng, Kui Zhang

**Affiliations:** 1 Department of Clinical Immunology, Xijing Hospital, Fourth Military Medical University, No.127 Changle West Road, Xi'an, 710032, China, 86 13572435012; 2 Department of Dermatology, The Air Force Hospital of Northern Theater PLA, Shenyang, China

**Keywords:** machine learning, knee osteoarthritis, progression, prediction model, meta-analysis

## Abstract

**Background:**

Machine learning (ML) has been investigated for its predictive value in knee osteoarthritis (KOA) progression. However, systematic evidence on the effectiveness of ML is still lacking, posing a challenge to precision prevention.

**Objective:**

This systematic review aimed to systematically assess the application status and accuracy of ML in predicting KOA progression and to compare the predictive performance of ML, traditional methods, and deep learning under different datasets, model types, modeling variables, and definitions of KOA progression.

**Methods:**

Following the PRISMA (Preferred Reporting Items for Systematic reviews and Meta-Analyses) statement, a systematic search was conducted in Embase, Web of Science, PubMed, and Cochrane Library up to October 10, 2025. Two investigators were independently responsible for study screening, data extraction, and risk-of-bias assessment in included studies using the Prediction Model Risk of Bias Assessment Tool. Meta-analyses were conducted on the concordance index (C-index) and diagnostic 4-fold table using a random effects model, with prediction intervals (PIs) reported. In addition, subgroup analyses were performed by model type, modeling variable, and definition of KOA progression.

**Results:**

A total of 32 studies were included. The overall risk of bias was considered low in 8 studies, high in 13 studies, and unclear in 11 studies. For predicting all progression, the pooled C-index was 0.773 (95% CI 0.727‐0.821; 95% PI 0.567‐1.000) for the clinical feature–based model, 0.798 (95% CI 0.755‐0.843; 95% PI 0.646‐0.984) for the magnetic resonance imaging (MRI)–based model, 0.712 (95% CI 0.657‐0.772; 95% PI 0.526‐0.965) for the X-ray–based model, 0.806 (95% CI 0.765‐0.849; 95% PI 0.639‐1.000) for the MRI+clinical feature–based model, 0.772 (95% CI 0.731‐0.815; 95% PI 0.610‐0.976) for the X-ray+clinical feature–based model, and 0.731 (95% CI 0.669‐0.798; 95% PI 0.518‐1.000) for the clinical feature+X-ray+MRI–based model. The clinical feature–based model was established mainly using logistic regression and exhibited accuracy comparable to other ML models. Among image-based models, traditional ML or deep learning possessed higher accuracy.

**Conclusions:**

This systematic review used CIs to estimate mean effects and PIs to estimate the potential range of effects in future scenarios. It systematically compared the performance of ML in predicting KOA progression under different model types, modeling variables, and definitions of KOA progression. ML models demonstrate certain discriminatory power in predicting KOA progression, but current evidence should be interpreted with caution due to various sources of significant heterogeneity, such as variations in the definition of KOA progression and validation strategies. Future research should standardize the definition of KOA progression, enhance methodological rigor, and conduct stringent external validation to improve model reliability and facilitate clinical translation.

## Introduction

Osteoarthritis (OA) is a degenerative joint disease characterized by degeneration of articular cartilage, osteophyte formation, and synovial inflammation, and it presents with pain, limitation of motion, and dysfunction [1]. OA is the 15th major cause of disability worldwide, with a prevalence rate of over 7% globally and up to 14% in high-income and aging countries. Moreover, it incurs health care costs that account for 1% to 2.5% of the gross domestic product of these countries [2]. As the most prevalent type of OA, knee OA (KOA) affects 365 million people, accounting for approximately 85% of the global burden of OA [3]. KOA has shown an annually increasing prevalence with population growth and aging, and it is projected that 642 million people will develop KOA globally by 2050, which underscores the urgency of active management strategies [4].

Despite rising economic costs, no specific treatment for KOA is available yet due to an unclear pathogenesis of KOA, and most patients remain at risk of KOA progression [5]. KOA progression primarily includes pain progression (an increase in the Western Ontario and McMaster Universities Osteoarthritis Index [WOMAC] pain subscale score) and imaging progression (a decline in joint space width [JSW] or an increase in Kellgren-Lawrence [KL] grade) [6,7]. Patients with advanced KOA need to undergo arthroplasty to restore joint function, but their postoperative functional outcome remains uncertain due to the limited lifespan of the prosthesis [8]. Therefore, early prediction of KOA progression is clinically important for developing specific preventive protocols.

The development of prediction tools for early progression encounters challenges due to the heterogeneity of structural and clinical features in KOA and therefore, few prediction tools for KOA progression are available [9]. Current clinical prediction models for KOA progression rely primarily on logistic regression models using demographic and basic clinical characteristics. However, these traditional statistical methods often fail to capture complex nonlinear interactions of high-dimensional risk factors, such as pixel-level texture variations in magnetic resonance imaging (MRI). Consequently, these traditional tools have limited capacity to handle complex, high-dimensional data despite their widespread use, and their predictive accuracy has reached a bottleneck, restricting their application in personalized medicine.

Machine learning (ML) can automatically learn complex nonlinear relationships and underlying patterns in data and exhibits greater adaptability and accuracy when handling high-dimensional unstructured data and large datasets. Therefore, research on ML techniques for predicting KOA progression has increasingly emerged [10]. Currently, ML, particularly deep learning (DL), has emerged as a powerful alternative. Unlike traditional methods requiring manual feature selection, DL algorithms can automatically extract potential features from raw medical images and capture subtle pathological changes that are often beyond human experts’ recognition capabilities and may be overlooked in conventional analysis. By integrating multimodal data, including clinical information, imaging data, and biomarkers, ML models can construct more comprehensive and accurate predictive systems for KOA progression. These systems enable more precise prediction and provide robust support for clinical decision-making.

Although the use of ML in KOA progression prediction has been summarized in narrative reviews [11-13], they have mostly conducted only qualitative analysis. Currently, a comprehensive evaluation of quantitative evidence regarding the discriminatory power (concordance index [C-index]) of these models is still lacking. Without considering the differences in study design and validation methods, it is difficult for meta-analyses to clearly determine whether ML possesses a more significant predictive advantage over traditional methods. Meanwhile, how to effectively integrate clinical data, imaging data, and multimodal data to optimize the predictive performance of ML models remains a critical issue that urgently needs to be addressed. In addition, outcome metrics for KOA progression may vary across studies, including the change in JSW, pain scores, or loss of function, making it more difficult to compare the study results.

Given variations in model types and modeling variables in available studies, the predictive effect of ML still requires systematic evidence. Therefore, this systematic review aimed to systematically assess the application status and predictive accuracy of ML models in KOA progression, quantitatively compare their performance with traditional statistical methods and DL, and assess the performance of ML models across subgroups (stratified by validation method, modeling variable, and definition of KOA progression). The findings are expected to provide an evidence-based basis for the future development of artificial intelligence prediction tools in this field.

## Methods

### Study Registration

To improve the reporting quality, this systematic review fully adhered to the PRISMA-DTA (Preferred Reporting Items for a Systematic Review and Meta-analysis of Diagnostic Test Accuracy) statement [14], and the PRISMA-DTA checklist is displayed in Checklist 1. The study protocol was registered with PROSPERO (CRD420251024340).

### Eligibility Criteria

The eligibility criteria are described in Textbox 1.

Textbox 1. **Inclusion and exclusion criteria.**

Inclusion criteria

- Patients diagnosed with knee osteoarthritis (KOA).
- ML models were established for predicting KOA progression (pain progression, imaging progression, and other progression).
- Outcome metrics were available for assessing model accuracy.
- Studies published in English.
- Studies with cohort, case-control, and cross-sectional designs.

Exclusion criteria

- Meeting abstracts not publicly available.
- Meta-analyses, reviews, guidelines, and expert opinions.
- Only risk factors were analyzed, without establishing complete machine learning (ML) models.
- Only image segmentation was performed, without establishing complete ML models.
- Only the association of a single factor with KOA progression was considered.
- Outcome metrics for assessing model accuracy were lacking.

### Data Source and Search Strategy

The process of study search followed the PRISMA-S (Preferred Reporting Items for Systematic reviews and Meta-Analyses–Search extension) guidelines to ensure transparency [15]. A systematic search was conducted in PubMed (National Library of Medicine), Embase (Elsevier), Web of Science (Clarivate Analytics), and Cochrane Library (Wiley), but we did not search specialized research registries or contact relevant experts for unpublished data. Additionally, the reference lists of all included studies were manually checked (backtracking) to avoid missing potentially relevant studies. The search strategy was developed by the first author and peer-reviewed by senior investigators in the team before final implementation. The search strategy used a combination of subject terms (Medical Subject Headings in PubMed and Emtree in Embase) and free-text keywords, primarily covering three key concepts: *knee osteoarthritis* (eg, “osteoarthritis,” “gonarthrosis”), *machine learning* (eg, “deep learning,” “random forest,” “Extreme Gradient Boosting [XGBoost]”), and *prediction or model* (eg, “prediction model,” “risk model”). Boolean operators (“AND” and “OR”) were also used to enhance search sensitivity. The search strategy for each database is provided in Supplementary Table S1 in Multimedia Appendix 1. No prepublished search filters or search strategies from other literature reviews were used, and no restrictions were imposed on language or study type. We reran the search formula to update the included studies, with the last search dated October 10, 2025.

### Study Selection

All retrieved records were imported into the literature management software EndNote (version 20; Clarivate). Duplicate publications were first removed by automatic deduplication (based on title, author, and year), followed by manual review to ensure accuracy. The initial search yielded 9631 records (PubMed: 1831, 19.0%; Embase: 4020, 41.7%; Web of Science: 2567, 26.7%; and Cochrane Library: 1213, 12.6%). After deduplication, 7161 (74.4%) records were incorporated into screening. Two investigators independently read the title and abstract and then examined the full text based on the eligibility criteria. Any discrepancy was resolved by discussion or adjudication by a third investigator.

### Data Extraction

A standardized spreadsheet was created to extract the following data: title, first author, year of publication, country, study type, patient source, type and definition of progression, duration of follow-up, number of progressive cases, total cases, number of progressive cases and total cases in the training and validation cohorts, generation method of the validation cohort, method for addressing overfitting, method for handling missing values, variables, model types, modeling variables, diagnostic 4-fold table, C-index, sensitivity, specificity, precision, accuracy, and F_1_-score. Any discrepancy was resolved by discussion or adjudication by a third investigator.

### Risk of Bias

The risk of bias (RoB) in the included studies was assessed using the Prediction Model Risk of Bias Assessment Tool [16]. The Prediction Model Risk of Bias Assessment Tool encompasses many questions across four domains: participants, predictors, outcome, and analysis, with each domain assessed for potential bias and applicability concerns. The signaling questions in each domain were answered as “yes or probably yes (low bias),” “no or probably no (high bias),” and “unclear.” The domain was deemed to be of high RoB if at least one question was rated as high bias and to be of low RoB if all questions were rated as low bias. Two investigators independently assessed RoB and cross-checked their results. Any discrepancy was resolved by adjudication by a third investigator.

### Data Synthesis

Stata (version 15.1; StataCorp) was used for meta-analyses, and the overall accuracy of the ML models was assessed by the C-index. The C-index is a measure of the consistency between the predicted risk score and the actual observed outcome; its value ranges from 0.5 to 1.0, with higher values indicating better discriminatory power of the model (0.5: predictions are equivalent to random guessing and 1.0: perfect prediction) [17]. When the SE with 95% CI for the C-index was missing in some studies, it was estimated using the methodology proposed by Debray et al [18]. Due to variations in variable screening strategies, variable optimization strategies, and variables across models, the models’ predictive performance may have potential differences. Therefore, a random effects model was used, and the prediction interval (PI) was calculated [19,20].

In addition, sensitivity and specificity underwent meta-analyses using bivariate mixed effects models based on diagnostic 4-fold tables. However, the diagnostic 4-fold table was not reported in most of the original studies, so it was calculated using sensitivity, specificity, precision, and the number of cases. Subgroup analyses were also performed by the dataset, model type, modeling variable, and definition of KOA progression.

## Results

### Search Results

**Figure 1.**
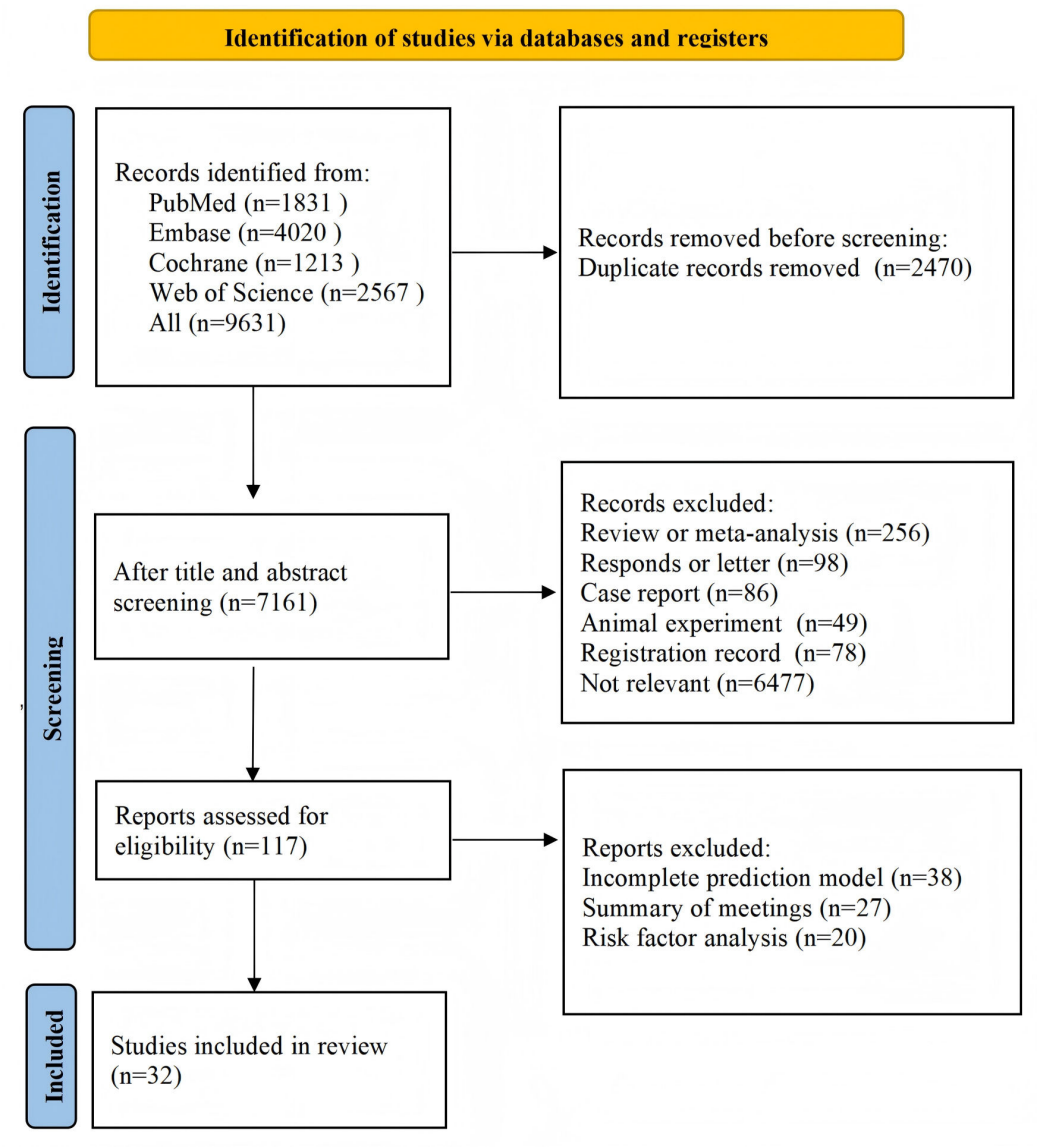
Study screening flowchart for systematic review of machine learning in predicting knee osteoarthritis progression.

Initially, 9631 studies were retrieved, of which 2470 (25.6%) duplicates were excluded. After the title and abstract review, 7044 (73.1%) irrelevant studies were excluded, including 256 (3.6%) meta-analyses or reviews, 98 (1.4%) letters or responses, 86 (1.2%) case reports, 49 (0.7%) in vitro trials, 78 (1.1%) registration protocols, and 6477 (92.0%) other irrelevant publications. The remaining 117 (1.2%) studies were examined for the full text. Finally, 38 (32.5%) studies lacking complete models, 27 (23.1%) conference abstracts, and 20 (17.1%) studies that only analyzed risk factors were excluded, and the remaining 32 (27.3%) studies were included, all of which had been published [7,21-51] (Figure 1).

### Study Characteristics

The included studies were all published between 2012 and 2025 and originated from China (n=12, 37.5%), the United States (n=6, 18.8%), Finland (n=4, 12.5%), France (n=2, 6.3%), Canada (n=2, 6.3%), the United Kingdom (n=1, 3.1%), Germany (n=1, 3.1%), Switzerland (n=1, 3.1%), Greece (n=1, 3.1%), Korea (n=1, 3.1%), and Australia (n=1, 3.1%). They were all observational studies, including 28 (87.5%) cohort studies and 4 (12.5%) case-control studies. The patient data were sourced from the Osteoarthritis Initiative database in 29 (90.6%) studies, from the Multicenter Osteoarthritis Study in 6 (18.8%) studies, and from other databases or a single center in 4 (12.5%) studies. A total of 20 (62.5%) studies reported imaging progression, 7 (21.9%) studies reported both pain progression and imaging progression, and 4 (12.5%) studies reported pain progression or dysfunction progression. The duration of follow-up greatly varied from 12 to 128 months. Thirty (93.8%) studies explicitly described the generation method of the validation cohort. Cross-validation was adopted in 23 (71.9%) studies, random sampling in 12 (37.5%) studies, and external validation in 5 (15.6%) studies. For modeling variables, clinical features were used in 23 (71.9%) studies, X-ray data were used in 17 (53.1%) studies, and MRI data were used in 18 (56.3%) studies. Logistic regression models were established in 9 (28.1%) studies, ML models in 20 (62.5%) studies, and image-based DL models in 16 (50.0%) studies (Table 1).

**Table 1.** Basic characteristics of the original studies included in this systematic review.

Author	Year	Country	Patient source	Study design	Progression type	Definition of progression	Follow-up duration (mo)	Progressive cases, n (%)	Total number of cases (N)	Generation method of the validation cohort	Cases in the validation cohort, n (%)	Model type	Modeling variable
Schiratti et al [43]	2021	France	OAI^a^	Retrospective cohort study	Imaging progression	JSW^b^ reduction ≥0.5 mm	12	806 (8.7)	9280	Cross-validation	N/A^c^	DL^d^	④^e^
Woloszynski et al [45]	2012	Australia	Single center	Retrospective cohort study	Imaging progression	Increase in the sum of JSN^f^ and osteophyte grades	48	59 (49.6)	119	N/A	N/A	KNN^g^	③^h^
Chan et al [25]	2021	China	OAI	Retrospective cohort study	Imaging progression, pain progression	Increase in WOMAC^i^ pain score ≥9 points, JSW reduction ≥0.7 mm	48	1183 (76.8)	1541	Cross-validation	N/A	LR^j^, MLP^k^, DT^l^	⑤^m^
Du et al [27]	2018	United States	OAI	Retrospective cohort study	Imaging progression	Increase in KL^n^ grade and JSN grade	24	N/A	100	Cross-validation	N/A	ANN^o^, SVM^p^, RF^q^, NB^r^	②^s^
Chen and Or [26]	2023	China	OAI	Retrospective cohort study	Pain progression, dysfunction progression	Increase in WOMAC pain and physical function scores	108	N/A	3200	Cross-validation	N/A	Weighted Ensemble, CatBoost^t^, Extra trees, LightGBM^u^, LightGBMXT, LightGBMLarge, XGBoost^v^, RF, KNN	③
Guan et al [30]	2022	United States	OAI	Retrospective cohort study	Pain progression	Increase in WOMAC pain score ≥9 points	48	2508 (50.2)	5000	Random sampling	500 (10.0)	ANN, DL	①^w^③⑤
Bayramoglu et al [23]	2024	Finland	MOST^x^	Retrospective cohort study	Imaging progression	Osteophyte score 2 or the JSN score 1 plus any osteophyte, sclerosis, or cysts 1 in the PF joint	84	403 (12.3)	3276	Cross-validation	N/A	GBM^y^, DL	①③⑤
Lee et al [38]	2025	Korea	OAI and MOST	Retrospective cohort study	Imaging progression	Increase in KL grade ≥1, with the increase in KL grade from 0 to 1 ignored	60	668 (11.2)	5966	External validation	3392 (56.9)	LR, KNN, GBM, RF, SVM, DT	⑤
Panfilov et al [40]	2025	Finland	OAI	Retrospective cohort study	Imaging progression	Increase in KL grade ≥1	96	670 (27.7)	2421	Cross-validation, random sampling	626 (25.9)	LR, DL	①②③⑥^z^
Yin et al [48]	2024	China	OAI	Retrospective cohort study	Imaging progression	Increase in KL grade ≥1, with the increase of KL grade from 0 to 1 ignored	48	964 (26.9)	3585	Cross-validation, random sampling	2653 (74.0)	DL	③
Jamshidi et al [35]	2020	Canada	OAI	Retrospective cohort study	Imaging progression	Increase in the percentage of the cartilage volume loss, KL grade ≥2, medial JSN ≥1	96	620 (38.8)	1598	Cross-validation	NA	LR, GBM, RF, MLP	⑥
Han et al [31]	2022	Germany	OAI and MOST	Retrospective cohort study	Imaging progression	Increase in KL grade ≥1	96	474 (9.9)	4796	Random sampling, external validation	2753 (57.4)	DL	③
Lv et al [39]	2025	China	OAI	Retrospective cohort study	Imaging progression, pain progression	Increase in WOMAC pain score ≥9 points, minimum medial JSW reduction ≥0.7 mm	48	194 (32.3)	600	Cross-validation, random sampling	120 (20.0)	SVM, RF, XGBoost, DL	②④
Joseph et al [37]	2022	United States	OAI	Retrospective cohort study	Imaging progression	KL progression from grade 0‐1 to grade 2‐4	96	183 (17.5)	1044	Cross-validation, random sampling	156 (14.9)	XGBoost	④
Jamshidi et al [34]	2025	Canada	OAI	Retrospective cohort study	Imaging progression	Based on 3 MRI features and 2 X-ray features, a threshold is applied to differentiate	NA	91 (59.9)	152	Cross-validation, random sampling	30 (19.7)	LR, ANN, DT, SVM, RF, GBM	①
Tiulpin et al [44]	2019	Finland	OAI and MOST	Retrospective cohort study	Imaging progression	Increase in KL grade	84	1331 (27.0)	4928	Cross-validation, external validation	3918 (79.5)	LR, GBM, DL	①③⑤
Dunn et al [29]	2023	United States	OABC^aa^+JoCoOA^ab^+OAI	Retrospective cohort study	Imaging progression, pain progression	Increase in WOMAC pain score ≥9 points, minimum JSW reduction ≥0.7 mm	48	365 (65.9)	554	Cross-validation, external validation	195 (35.2)	LR	①
Ashinsky et al [22]	2017	United States	OAI	Retrospective cohort study	Symptom progression	Increase in WOMAC pain score ≥10	36	40 (58.8)	68	N/A	N/A	KNN	②
Panfilov et al [41]	2022	Finland	OAI	Retrospective cohort study	Imaging progression	Increase in KL grade	96	1315 (27.0)	4866	Cross-validation	1259 (25.9)	DL	②
Castagno et al [24]	2025	United Kingdom	OAI and POMA^ac^	Retrospective cohort study	Imaging progression, pain progression	Increase in WOMAC pain score ≥2 points, minimum medial JSW reduction ≥0.6 mm, or KL grade =4	24	666 (39.4)	1691	Cross-validation, random sampling, external validation	705 (41.7)	XGBoost, CatBoost, LR, LGBM, RF	①②⑤⑥
Salis et al [42]	2024	Switzerland	OAI and MOST	Retrospective cohort study	Imaging progression, pain progression	Total WOMAC pain and dysfunction score ≥12 points, with a KL grade of 4; or the total WOMAC pain and dysfunction score ≥23 points, with a KL grade of 2 or 3	60	859 (23.1)	3720	External validation	1602 (43.1)	XGBoost	⑤
Almhdie-Imjabbar et al [21]	2022	France	OAI and MOST	Retrospective cohort study	Imaging progression	Increase in medial JSN grade	60	228 (13.8)	1647	Cross-validation, external validation	376 (22.8)	DL	⑤
Xiao et al [46]	2021	China	OAI	Retrospective cohort study	Pain progression, dysfunction progression, stiffness progression, symptom progression	Increase in WOMAC pain score	12	N/A	551	Random sampling	151 (27.4)	LR, NB, KNN, SVM, RF	⑥
Xing et al [47]	2025	China	TASOAC^ad^	Retrospective cohort study	Pain progression, dysfunction progression, imaging progression	≥1% per year loss in cartilage volume	128	240 (41.8)	574	Cross-validation	N/A	LR, GBM	④
Cheung et al [7]	2021	China	OAI	Retrospective cohort study	Imaging progression	Increase in KL grade	48	N/A	945	Cross-validation	N/A	DL	③
Du et al [28]	2018	United States	OAI	Retrospective cohort study	Imaging progression	Increase in KL grade and JSN grade	24	N/A	200	Cross-validation	N/A	ANN	②
Theocharis et al [50]	2025	Greece	OAI	Retrospective cohort study	Imaging progression	Increase in KL grade	48	N/A	6228	Cross-validation	N/A	DL	④
Wang et al [51]	2025	China	OAI	Retrospective cohort study	Imaging progression, pain progression	Increase in WOMAC pain score ≥9 points, minimum JSW reduction ≥0.7 mm	24	194 (32.7)	594	Cross-validation, random sampling	297 (50.0)	DL	④
Hu et al [33]	2023	China	OAI	Retrospective case-control study	Imaging progression, pain progression	Increase in WOMAC pain score ≥9 points, minimum medial JSW reduction ≥0.7 mm	48	182 (50.0)	364	Cross-validation	N/A	MLP, DL	①②③④
Jiang et al [36]	2024	China	OAI	Retrospective case-control study	Imaging progression	Minimum medial JSW reduction .7 mm	24	289 (51.2)	565	Random sampling	170 (30.1)	SVM	①②
Yu et al [49]	2023	China	OAI	Retrospective case-control study	Imaging progression	KL grade ≥2 during the follow-up visit	48	302 (50.0)	604	Cross-validation, random sampling	242 (40.1)	DL	①②④
Hu et al [32]	2025	China	OAI	Retrospective case-control study	Imaging progression	Minimum medial JSW reduction ≥0.7 mm	24	194 (32.3)	600	Random sampling	120 (20.0)	DL	①②④

a OAI: osteoarthritis Initiative database.

b JSW: joint space width.

c N/A: not applicable.

d DL: deep learning.

e ④: magnetic resonance imaging (MRI)+clinical features.

f JSN: joint space narrowing.

g KNN: K-nearest neighbor.

h ③: X-ray features.

i WOMAC: Western Ontario and McMaster Universities Osteoarthritis Index.

j LR: logistic regression.

k MLP: multilayer perceptron.

l DT: decision tree.

m ⑤: X-ray+clinical features

n KL: Kellgren-Lawrence.

o ANN: artificial neural network.

p SVM: support vector machine.

q RF: random forest.

r NB: naive Bayes.

s ②: MRI features.

t CatBoost: category gradient-boosting.

u LightGBM: Light Gradient Boosting Machine.

v XGBoost: eXtreme Gradient Boosting.

w ①: clinical features.

x MOST: Multicenter Osteoarthritis Study.

y GBM: Gradient Boosting Machine.

z ⑥: X-Ray+MRI+clinical features.

aa OABC: Osteoarthritis Biomarkers Consortium.

ab JoCoOA: Johnston County Osteoarthritis Project.

ac POMA: Pivotal Osteoarthritis Initiative MRI Analyses.

ad TASOAC: Tasmania Older Adult Cohort.

### Risk of Bias

**Figure 2.**
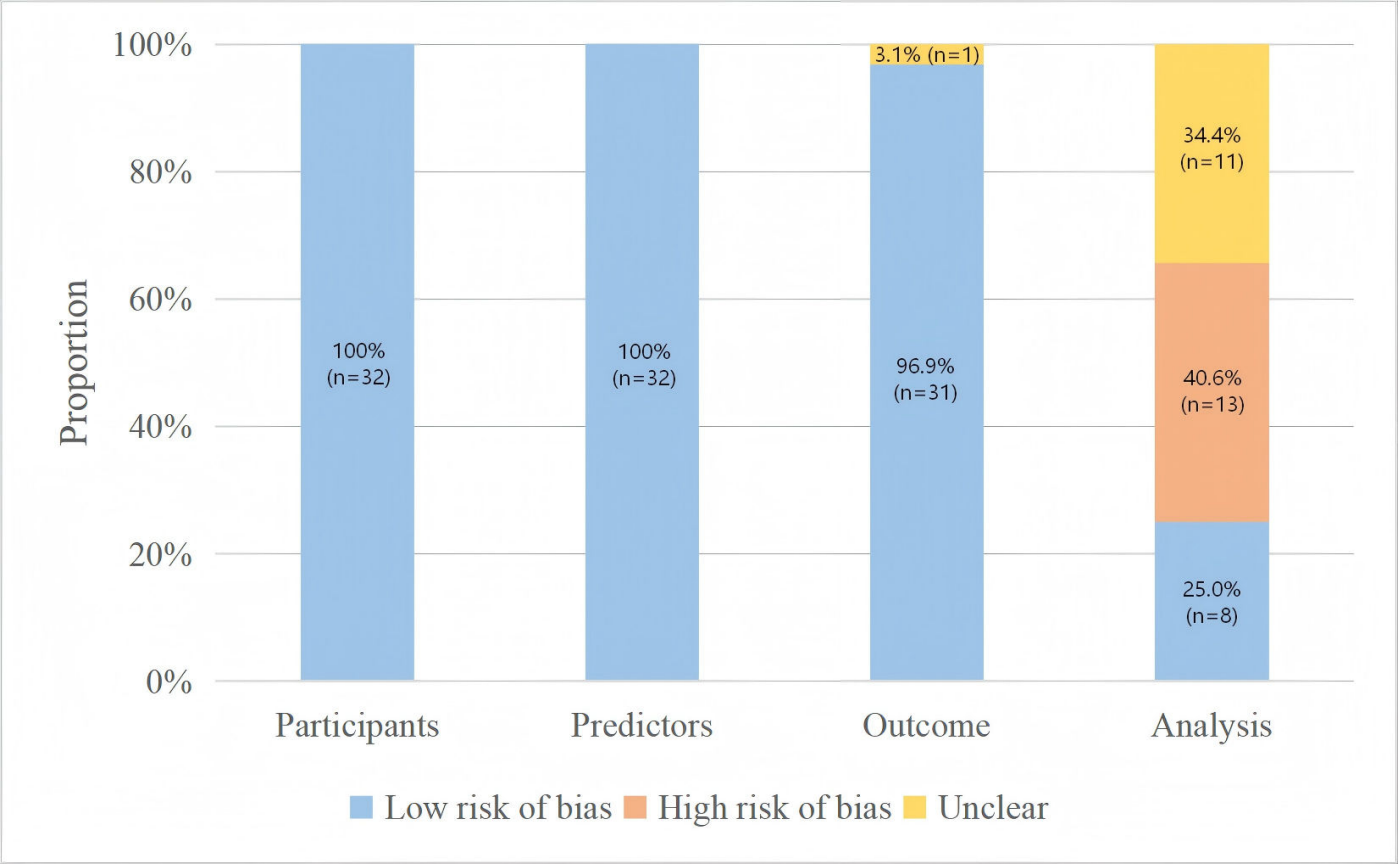
Summary of risk of bias assessment results by Prediction Model Risk of Bias Assessment Tool for included original studies.

The overall RoB was considered low in 8 (25.0%) studies, high in 13 (40.6%) studies, and unclear in 11 (34.4%) studies. Specifically, all studies had a low risk in the domains of participants and predictors; in the domain of outcome, 31 (96.9%) studies had low risk, and 1 (3.1%) had unclear risk; in the analysis domain, 8 (25.0%) studies had low risk, 13 (40.6%) studies had high risk, and 11 (34.4%) studies had unclear risk (Figure 2 and Table S2 in Multimedia Appendix 1).

### Meta-Analysis

#### All Progression

**Figure 3.**
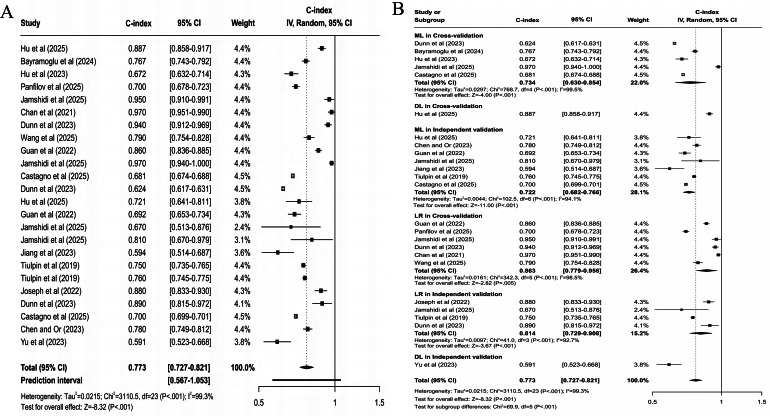
Forest plot for meta-analysis of C-index of clinical feature–based model for predicting all progression of knee osteoarthritis [23-26,29,30,32-34,36,37,40,44,49,51]. (A) Main meta-analysis; (B) subgroup analysis. DL: deep learning; LR: logistic regression; ML: machine learning.

**Figure 4.**
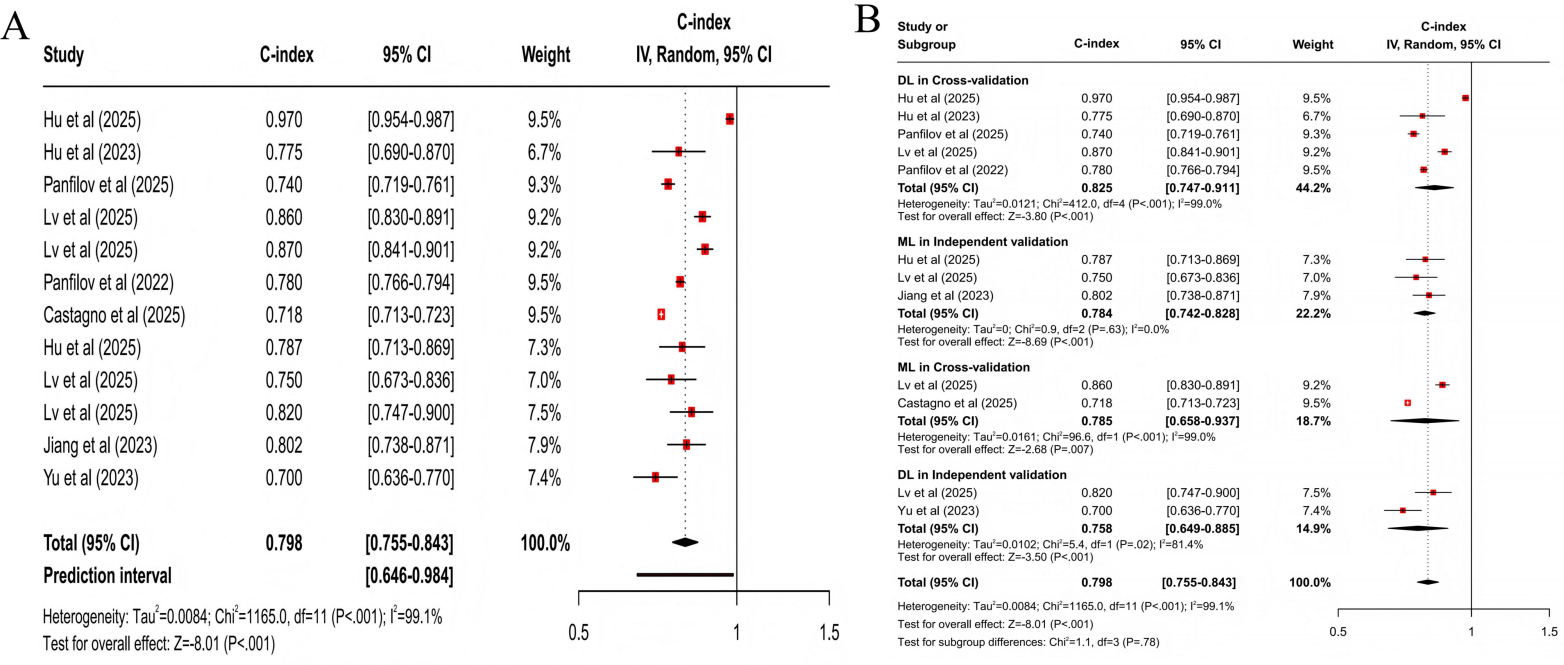
Forest plot for meta-analysis of C-index of MRI-based model for predicting all progression of knee osteoarthritis [24,32,33,36,39-41,49]. (A) Main meta-analysis; (B) subgroup analysis. DL: deep learning; LR: logistic regression; ML: machine learning.

**Figure 5.**
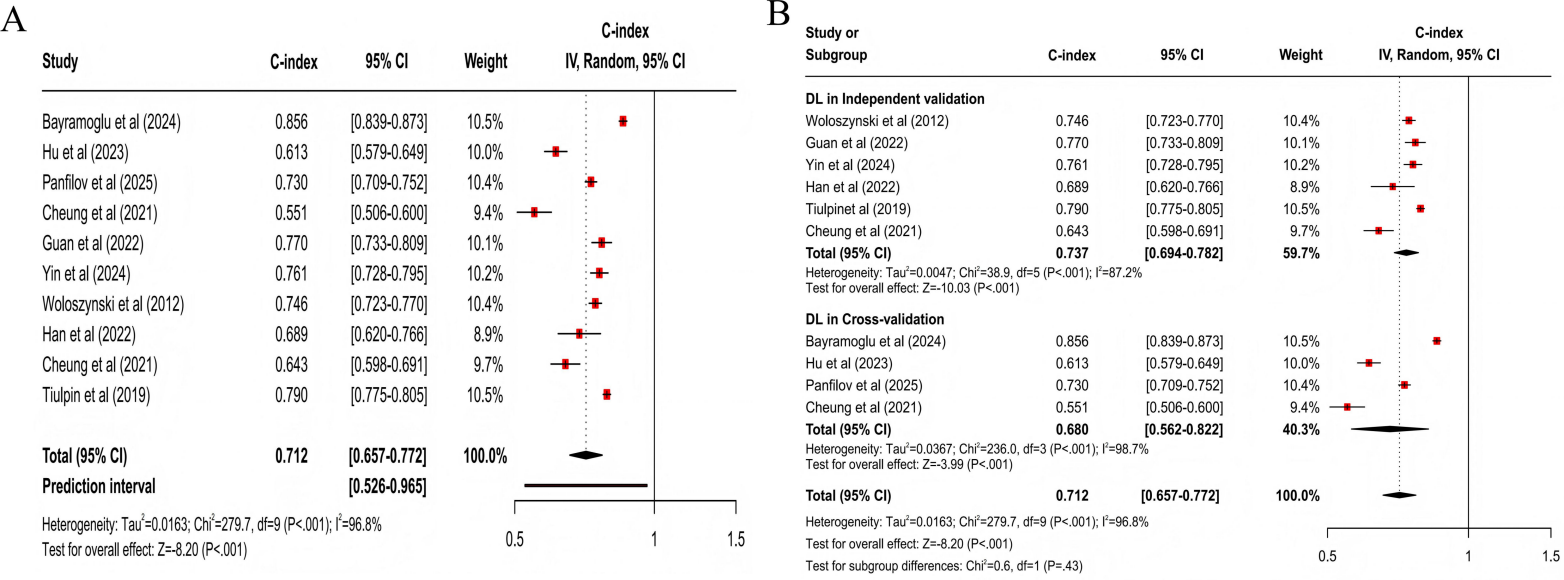
Forest plot for meta-analysis of C-index of X-ray–based model for predicting all progression of knee osteoarthritis [7,23,30,31,33,40,44,45,48]. (A) Main meta-analysis; (B) subgroup analysis. DL: deep learning; LR: logistic regression; ML: machine learning.

**Figure 6.**
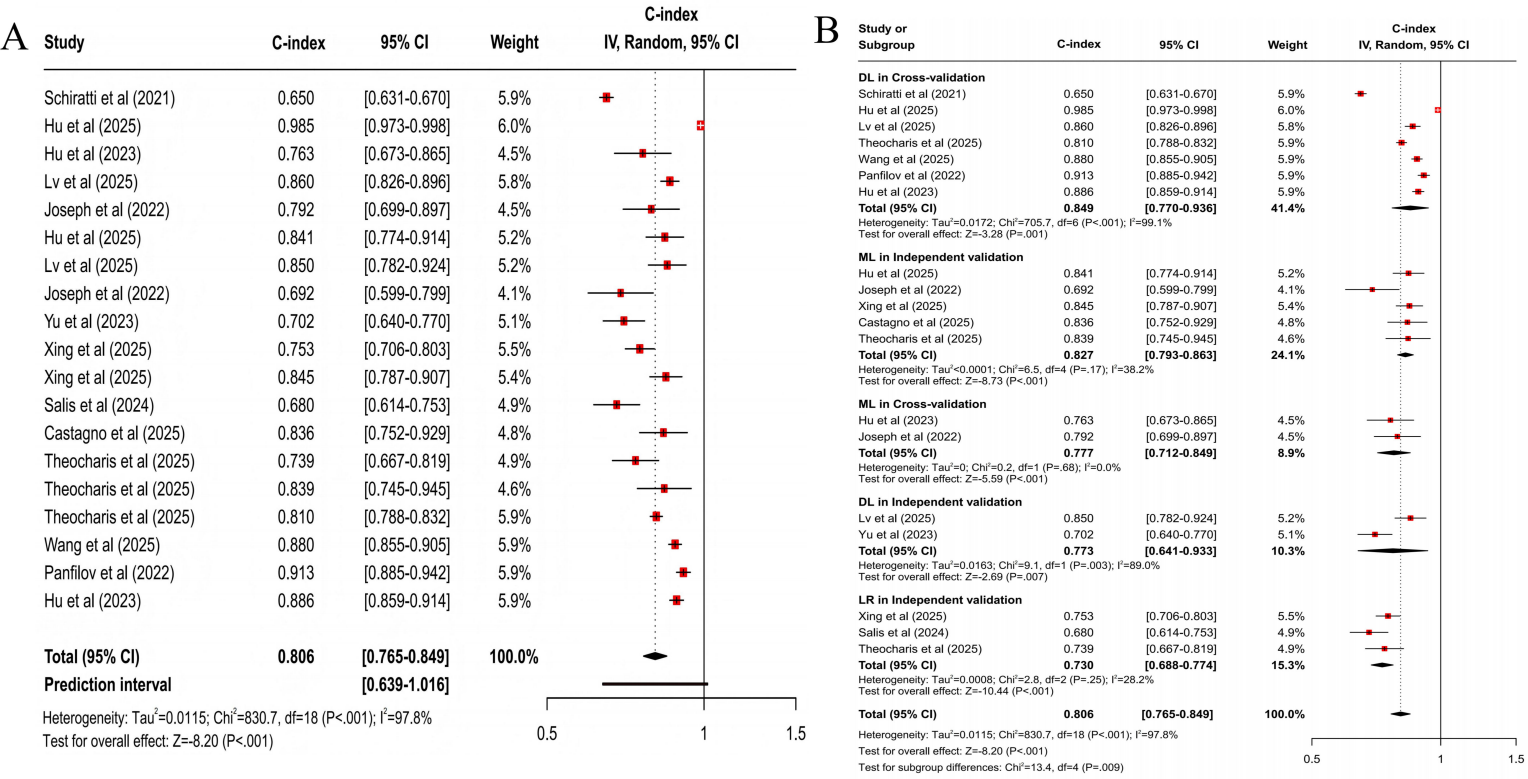
Forest plot for meta-analysis of C-index of MRI+clinical feature–based model for predicting all progression of knee osteoarthritis [24,32,33,37,39,41-43,47,49-51]. (A) Main meta-analysis; (B) subgroup analysis. DL: deep learning; LR: logistic regression; ML: machine learning.

**Figure 7.**
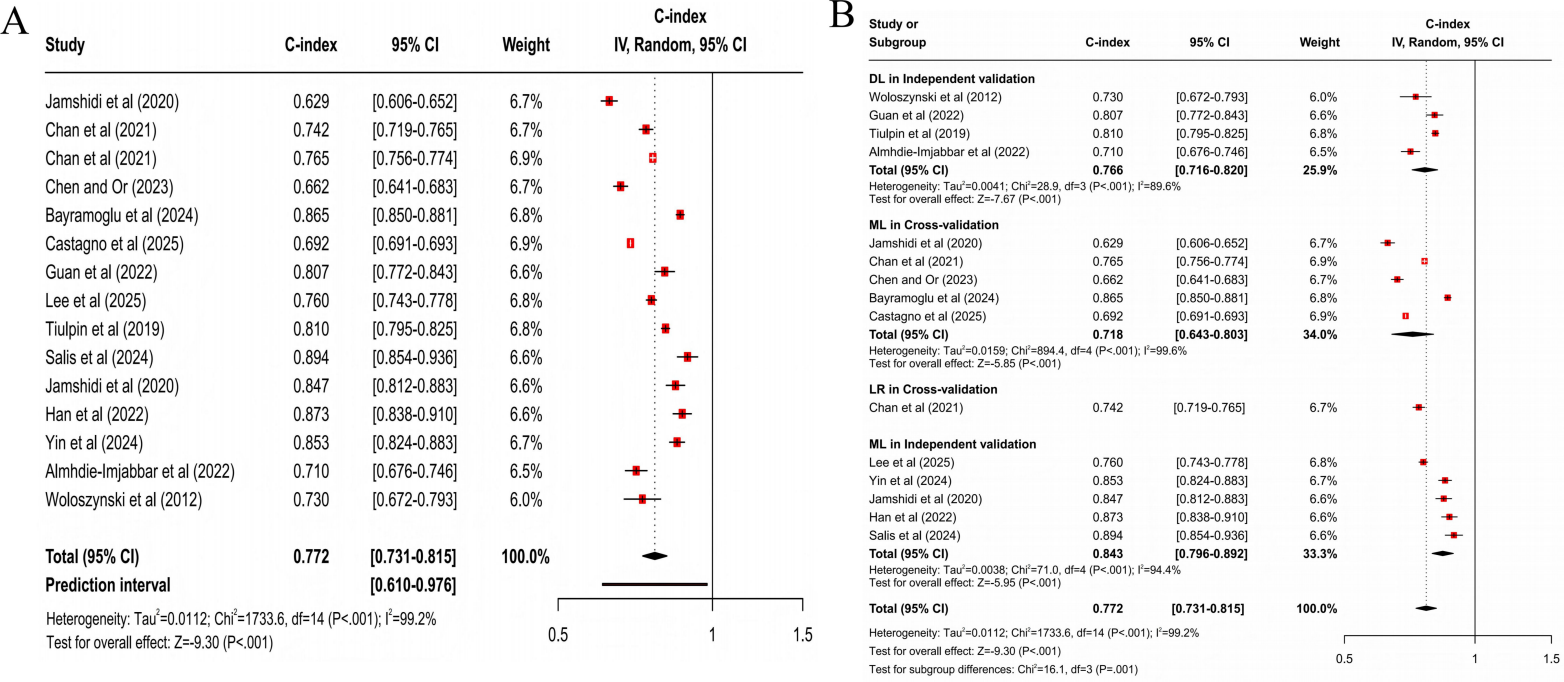
Forest plot for meta-analysis of C-index of X-ray+clinical feature–based model for predicting all progression of knee osteoarthritis [21,23-26,30,31,35,38,42,44,45,48]. (A) Main meta-analysis; (B) subgroup analysis. DL: deep learning; LR: logistic regression; ML: machine learning.

**Figure 8.**
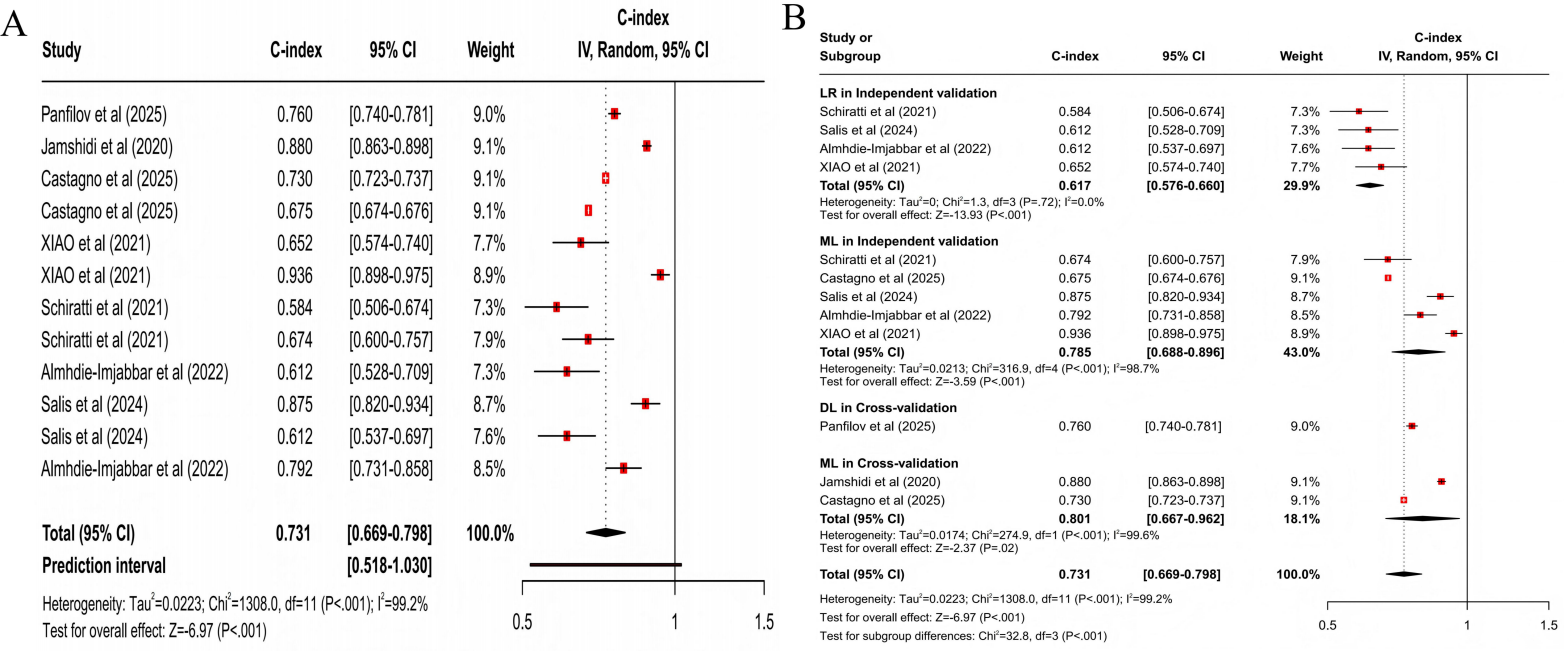
Forest plot for meta-analysis of C-index of clinical feature+X-ray+MRI–based model for predicting all progression of knee osteoarthritis [21,24,35,40,42,43,46]. (A) Main meta-analysis; (B) subgroup analysis. DL: deep learning; LR: logistic regression; ML: machine learning.

For predicting all progression, the clinical feature–based model had a pooled C-index of 0.773 (95% CI 0.727‐0.821; 95% PI 0.567‐1.000; *z* score=−8.32; *P*<.001; Figure 3A), with sensitivity and specificity of 0.77 (95% CI 0.68‐0.84) and 0.75 (95% CI 0.67‐0.81), respectively; the MRI-based model had a pooled C-index of 0.798 (95% CI 0.755‐0.843; 95% PI 0.646‐0.984; *z* score=−8.01; *P*<.001; Figure 4A), with sensitivity and specificity of 0.75 (95% CI 0.68‐0.81) and 0.77 (95% CI 0.74‐0.80); the X-ray–based model had a pooled C-index of 0.712 (95% CI 0.657‐0.772; 95% PI 0.526‐0.965; *z* score=−8.20; *P*<.001; Figure 5A), with sensitivity and specificity of 0.72 (95% CI 0.67‐0.76) and 0.70 (95% CI 0.64‐0.75), respectively; the MRI+clinical feature–based model had a pooled C-index of 0.806 (95% CI 0.765‐0.849; 95% PI 0.639‐1.000; *z* score=−8.20; *P*<.001; Figure 6A), with sensitivity and specificity of 0.77 (95% CI 0.70‐0.83) and 0.77 (95% CI 0.71‐0.82), respectively; the X-ray+clinical feature–based model had a pooled C-index of 0.772 (95% CI 0.731‐0.815; 95% PI 0.610‐0.976; *z* score=−9.30; *P*<.001; Figure 7A), with sensitivity and specificity of 0.72 (95% CI 0.67‐0.77) and 0.76 (95% CI 0.72‐0.80), respectively; the clinical feature+X-ray+MRI–based model had a pooled C-index of 0.731 (95% CI 0.669‐0.798; 95% PI 0.518‐1.000; *z* score=−6.97; *P*<.001; Figure 8A), with sensitivity and specificity of 0.68 (95% CI 0.57‐0.78) and 0.74 (95% CI 0.64‐0.82), respectively.

In the aforementioned main meta-analysis, the PIs for all pooled C-indices were broad, with lower limits below 0.7 (a C-index >0.7 suggests satisfactory discriminatory power of the model). Therefore, subgroup analyses were conducted. Subgroup analyses were conducted by the validation method (ie, cross-validation and independent validation). The results revealed that the pooled C-index of X-ray–based and X-ray+clinical feature–based models was superior in independent validation to that in cross-validation, while the pooled C-index of clinical feature–, MRI-, MRI+clinical feature–, or clinical feature+X-ray+MRI–based models was superior in cross-validation to that in independent validation. Under different modeling variables, subgroup analyses were also performed by the modeling method (logistic regression, traditional ML, and DL) to pool the accuracy of these models in predicting disease progression (Tables2  3).

**Table 2.** Meta-analysis of C-index of machine learning (ML) for predicting all progression of knee osteoarthritis.

Modeling variables and model	Cross-validation					Independent validation					Overall				
	n (%)	C-index (95% CI)	PI^a^	τ	τ²	n (%)	C-index (95% CI)	PI	τ	τ²	n (%)	C-index (95% CI)	PI	τ	τ²
Clinical features															
LR^b^	6 (50.0)	0.863 (0.779‐0.956)	—^c^	0.1269	0.0161	4 (33.3)	0.814 (0.729‐0.908)	—	0.0987	0.0097	10 (41.7)	0.844 (0.783‐0.910)	0.642‐1.000	0.1151	0.0132
ML	5 (41.7)	0.734 (0.630‐0.854)	—	0.1723	0.0297	7 (58.4)	0.722 (0.682‐0.766)	—	0.0663	0.0044	12 (50.0)	0.726 (0.675‐0.781)	0.547‐0.964	0.1234	0.0152
DL^d^	1 (8.3)	0.887 (0.858‐0.917)	—	—	—	1 (8.3)	0.591 (0.519‐0.663)	—	—	—	2 (8.3)	0.727 (0.489‐1.000)	—	0.2834	0.0803
Overall	12	0.808 (0.738‐0.885)	0.561‐1.000	0.1595	0.0254	12	0.737 (0.686‐0.792)	0.563‐0.964	0.1163	0.0135	24	0.773 (0.727‐0.821)	0.567‐1.000	0.1465	0.0215
MRI^e^ features															
LR	—	—	—	—	—	—	—	—	—	—	—	—	—	—	—
ML	2 (28.6)	0.785 (0.658‐0.937)	—	0.1269	0.0161	3 (60.0)	0.784 (0.742‐0.828)	—	0	0	5 (41.7)	0.782 (0.729‐0.840)	0.626‐0.978	0.0719	0.0052
DL	5 (71.4)	0.825 (0.747‐0.911)	—	0.1098	0.0121	2 (40.0)	0.758 (0.649‐0.885)	—	0.1009	0.0102	7 (58.3)	0.807 (0.743‐0.877)	0.609‐1.000	0.1068	0.0114
Overall	7	0.813 (0.750‐0.882)	0.615‐1.000	0.1067	0.0114	5	0.772 (0.730‐0.817)	0.672‐0.888	0.0413	0.0017	12	0.798 (0.755‐0.843)	0.646‐0.984	0.0914	0.0084
X-ray features															
LR	—	—	—	—	—	—	—	—	—	—	—	—	—	—	—
ML	—	—	—	—	—	—	—	—	—	—	—	—	—	—	—
DL	4 (100)	0.680 (0.562‐0.822)	—	0.1916	0.0367	6 (100)	0.737 (0.694‐0.782)	—	0.0687	0.0047	10 (100)	0.712 (0.657‐0.772)	0.526‐0.965	0.1276	0.0163
Overall	4	0.680 (0.562‐0.822)	0.343‐1.000	0.1916	0.0367	6	0.737 (0.694‐0.782)	0.607‐0.894	0.0687	0.0047	10	0.712 (0.657‐0.772)	0.526‐0.965	0.1276	0.0163
MRI+clinical features															
LR	—	—	—	—	—	3 (30.0)	0.730 (0.688‐0.774)	—	0.0284	0.0008	3 (15.8)	0.730 (0.688‐0.774)	0.611‐0.872	0.0284	0.0008
ML	2 (22.2)	0.777 (0.712‐0.849)	—	0	0	5 (50.0)	0.827 (0.793‐0.863)	—	0.0200	0.0004	7 (36.8)	0.816 (0.783‐0.850)	0.762‐0.873	0.0180	0.0003
DL	7 (77.8)	0.849 (0.770‐0.936)	—	0.1312	0.0172	2 (20.0)	0.773 (0.641‐0.933)	—	0.1276	0.0163	9 (47.4)	0.833 (0.765‐0.907)	0.609‐1.000	0.1285	0.0165
Overall	9	0.835 (0.770‐0.906)	0.622‐1.000	0.1211	0.0147	10	0.778 (0.735‐0.823)	0.647‐0.935	0.0760	0.0058	19	0.806 (0.765‐0.849)	0.639‐1.000	0.1071	0.0115
X-ray+clinical features															
LR	1 (16.7)	0.742 (0.719‐0.765)	—	—	—	—	—	—	—	—	1 (6.7)	0.742 (0.719‐0.765)	—	—	—
ML	5 (83.3)	0.718 (0.643‐0.803)		0.1259	0.0159	5 (55.6)	0.843 (0.796‐0.892)		0.0614	0.0038	10 (66.7)	0.778 (0.719‐0.842)	0.577‐1.000	0.1256	0.0158
DL	—	—	—	—	—	4 (44.4)	0.766 (0.716‐0.820)	—	0.0643	0.0041	4 (26.6)	0.766 (0.716‐0.820)	0.607‐0.967	0.0643	0.0041
Overall	6	0.722 (0.659‐0.791)	0.527‐1.000	0.1133	0.0128	9	0.809 (0.768‐0.851)	0.673‐0.971	0.0750	0.0056	15	0.772 (0.731‐0.815)	0.610‐0.976	0.1060	0.0112
Clinical feature+X-ray+MRI															
LR	—	—	—	—	—	4 (44.4)	0.617 (0.576‐0.660)	—	0	0	4 (33.3)	0.617 (0.576‐0.660)	0.552‐0.689	0	0
ML	2 (66.7)	0.801 (0.667‐0.962)	—	0.1319	0.0174	5 (55.6)	0.785 (0.688‐0.896)	—	0.1460	0.0213	7 (58.4)	0.790 (0.716‐0.873)	0.562‐0.976	0.1300	0.0169
DL	1 (33.3)	0.760 (0.740‐0.780)	—	—	—	—	—	—	—	—	1 (8.3)	0.760 (0.740‐0.780)	—	—	—
Overall	3	0.787 (0.704‐0.880)	0.483‐1.000	0.098	0.0096	9	0.709 (0.634‐0.793)	0.476‐1.000	0.1631	0.0266	12	0.731 (0.669‐0.798)	0.518‐1.000	0.1494	0.0223

a PI: prediction interval.

b LR: logistic regression.

c Not available.

d DL: deep learning.

e MRI: magnetic resonance imaging.

**Table 3.** Meta-analysis of sensitivity and specificity of machine learning (ML) for predicting all progression of knee osteoarthritis.

Modeling variables and model	Cross-validation			Independent validation			Overall		
	n (%)	Sensitivity (95% CI)	Specificity (95% CI)	n (%)	Sensitivity (95% CI)	Specificity (95% CI)	n (%)	Sensitivity (95% CI)	Specificity (95% CI)
Clinical features									
LR^a^	4 (44.4)	0.94 (0.72‐0.99)	0.81 (0.68‐0.89)	4 (33.3)	0.72 (0.65‐0.79)	0.78 (0.67‐0.87)	8 (38.1)	0.85 (0.68‐0.94)	0.81 (0.73‐0.87)
ML	4 (44.4)	0.77 (0.59‐0.88)	0.75 (0.66‐0.83)	7 (58.4)	0.68 (0.52‐0.80)	0.69 (0.49‐0.84)	11 (52.4)	0.71 (0.59‐0.80)	0.71 (0.58‐0.81)
DL^b^	1 (11.2)	0.80	0.84	1 (8.3)	0.64	0.42	2 (9.5)	0.64‐0.80	0.42‐0.84
Overall	9	0.86 (0.74‐0.93)	0.79 (0.72‐0.85)	12	0.67 (0.58‐0.76)	0.71 (0.57‐0.81)	21	0.77 (0.68‐0.84)	0.75 (0.67‐0.81)
MRI^c^ features									
LR	—^d^	—	—	—	—	—	—	—	—
ML	2 (33.3)	0.67‐0.80	0.78‐0.79	3 (60.0)	0.77‐0.83	0.63‐0.80	5 (45.5)	0.77 (0.70‐0.83)	0.74 (0.69‐0.79)
DL	4 (66.7)	0.79 (0.66‐0.88)	0.78 (0.73‐0.82)	2 (40.0)	0.53‐0.62	0.80‐0.83	6 (54.5)	0.74 (0.61‐0.84)	0.77 (0.73‐0.80)
Overall	6	0.77 (0.68‐0.85)	0.78 (0.75‐0.81)	5	0.71 (0.59‐0.81)	0.75 (0.67‐0.81)	11	0.75 (0.68‐0.81)	0.77 (0.74‐0.80)
X-ray features									
LR	—	—	—	—	—	—	—	—	—
ML	—	—	—	—	—	—	—	—	—
DL	1 (100)	0.79	0.76	6 (100)	0.70 (0.65‐0.75)	0.68 (0.63‐0.74)	7 (100)	0.72 (0.67‐0.76)	0.70 (0.64‐0.75)
Overall	1	0.79	0.76	6	0.70 (0.65‐0.75)	0.68 (0.63‐0.74)	7	0.72 (0.67‐0.76)	0.70 (0.64‐0.75)
MRI+clinical features									
LR	—	—	—	3 (30.0)	0.65‐0.81	0.60‐0.69	3 (16.7)	0.65‐0.81	0.60‐0.69
ML	2 (25.0)	0.37‐0.66	0.76‐0.90	5 (50.0)	0.75 (0.62‐0.85)	0.79 (0.73‐0.84)	7 (38.9)	0.68 (0.54‐0.79)	0.80 (0.74‐0.85)
DL	6 (75.0)	0.85 (0.80‐0.89)	0.79 (0.65‐0.89)	2 (20.0)	0.64‐0.85	0.69‐0.70	8 (44.4)	0.83 (0.76‐0.88)	0.77 (0.65‐0.86)
Overall	8	0.79 (0.67‐0.88)	0.80 (0.70‐0.88)	10	0.74 (0.67‐0.81)	0.73 (0.68‐0.78)	18	0.77 (0.70‐0.83)	0.77 (0.71‐0.82)
X-ray+clinical features									
LR	1 (16.7)	0.66	0.80	—	—	—	1 (9.1)	0.66	0.80
ML	5 (83.3)	0.70 (0.61‐0.78)	0.75 (0.69‐0.81)	3 (60.0)	0.72‐0.89	0.65‐0.83	8 (72.7)	0.73 (0.66‐0.79)	0.76 (0.70‐0.81)
DL	—	—	—	2 (40.0)	0.72‐0.75	0.72‐0.81	2 (18.2)	0.72‐0.75	0.72‐0.81
Overall	6	0.69 (0.61‐0.76)	0.76 (0.70‐0.81)	5	0.75 (0.72‐0.77)	0.77 (0.70‐0.82)	11	0.72 (0.67‐0.77)	0.76 (0.72‐0.80)
Clinical feature+X-ray+MRI									
LR	—	—	—	4 (44.4)	0.58 (0.41‐0.72)	0.63 (0.48‐0.76)	4 (36.4)	0.58 (0.41‐0.72)	0.63 (0.48‐0.76)
ML	2 (100)	0.68‐0.87	0.79‐0.90	5 (55.6)	0.71 (0.53‐0.84)	0.75 (0.61‐0.85)	7 (63.6)	0.74 (0.60‐0.84)	0.79 (0.68‐0.87)
DL	—	—	—	—	—	—	—	—	—
Overall	2	0.68‐0.87	0.79‐0.90	9	0.65 (0.52‐0.76)	0.70 (0.59‐0.79)	11	0.68 (0.57‐0.78)	0.74 (0.64‐0.82)

a LR: logistic regression;

b DL: deep learning.

c MRI: magnetic resonance imaging.

d Not available.

Differences were statistically significant in clinical feature–based models across subgroups (χ^2_5_^=69.9; *P*<.001; Figure 3B). When cross-validation was used, the logistic regression model demonstrated higher accuracy, with a pooled C-index of 0.863 (95% CI 0.779‐0.956; *z* score=−2.82; *P*=.005). When independent validation was used, the logistic regression model also displayed higher accuracy, with a pooled C-index of 0.814 (95% CI 0.729‐0.908; *z* score=−3.67; *P*<.001). Besides, differences were not statistically significant in MRI-based models across subgroups (*χ*^2_3_^=1.1; *P*=.78; Figure 4B). When cross-validation was used, the DL model demonstrated higher accuracy, with a pooled C-index of 0.825 (95% CI 0.747‐0.911; *z* score=−3.80; *P*<.001). When independent validation was used, the ML model displayed optimal predictive performance, with a pooled C-index of 0.784 (95% CI 0.742‐0.828; *z* score=−8.69; *P*<.001). Differences were not statistically significant in X-ray–based models across subgroups (*χ*^2_1_^=0.6; *P*=.43; Figure 5B). When cross-validation was used, only DL models were included, yielding lower mean predictive power, with a C-index of 0.680 (95% CI 0.562‐0.822; *z* score=−3.99; *P*<.001). When independent validation was used, only DL models were included and displayed higher accuracy, with a pooled C-index of 0.737 (95% CI 0.694‐0.782; *z* score=−10.03; *P*<.001).

For multimodal models, statistically significant differences were present in MRI+clinical feature–based models across subgroups (*χ*^2_4_^=13.4; *P*=.009; Figure 6B). When cross-validation was used, the DL model demonstrated superior accuracy, with a pooled C-index of 0.849 (95% CI 0.770‐0.936; *z* score=−3.28; *P*=.001). When independent validation was used, the ML model demonstrated higher accuracy, with a C-index of 0.827 (95% CI 0.793‐0.863; *z* score=−8.73; *P*<.001). Besides, statistically significant differences were present in X-ray+clinical feature–based models (*χ*^2_3_^=16.1; *P*=.001; Figure 7B). When cross-validation was used, the ML model yielded a C-index of 0.718 (95% CI 0.643‐0.803; *z* score=−5.85; *P*<.001). When independent validation was used, the ML model demonstrated higher accuracy, with a C-index of 0.843 (95% CI 0.796‐0.892; *z* score=−5.95; *P*<.001). Clinical feature+X-ray+MRI–based models had statistically significant differences across subgroups (*χ*^2_3_^=32.8; *P*<.001; Figure 8B). When cross-validation was used, the ML model yielded a C-index of 0.801 (95% CI 0.667‐0.962; *z* score=−2.37; *P*=.02). When independent validation was used, the ML model also demonstrated higher accuracy, with a pooled C-index of 0.785 (95% CI 0.688‐0.896; *z* score=−3.59; *P*<.001).

#### Imaging Progression

For predicting imaging progression, the clinical feature–based model had a pooled C-index of 0.791 (95% CI 0.730‐0.857; 95% PI 0.566‐1.000; *z* score=−5.74; *P*<.001; Figure S1A in Multimedia Appendix 1), with sensitivity and specificity of 0.81 (95% CI 0.72‐0.88) and 0.71 (95% CI 0.60‐0.80); the MRI-based model had a pooled C-index of 0.795 (95% CI 0.725‐0.872; 95% PI 0.584‐1.000; *z* score=−4.87; *P*<.001; Figure S2A in Multimedia Appendix 1), with sensitivity and specificity of 0.76 (95% CI 0.62‐0.86) and 0.78 (95% CI 0.72‐0.83); the X-ray–based model had a pooled C-index of 0.718 (95% CI 0.655‐0.788; 95% PI 0.518‐0.997; *z* score=−7.01; *P*<.001; Figure S3A in Multimedia Appendix 1), with sensitivity and specificity of 0.71 (95% CI 0.65‐0.76) and 0.69 (95% CI 0.63‐0.75); the MRI+clinical feature–based model had a pooled C-index of 0.796 (95% CI 0.732‐0.865; 95% PI 0.586‐1.000; *z* score=−5.39; *P*<.001; Figure S4A in Multimedia Appendix 1), with sensitivity and specificity of 0.77 (95% CI 0.63‐0.87) and 0.78 (95% CI 0.66‐0.86); the X-ray+clinical feature–based model had a pooled C-index of 0.748 (95% CI 0.684‐0.818; 95 % PI 0.550‐1.000; *z* score=−6.32; *P*<.001; Figure S5A in Multimedia Appendix 1), with sensitivity and specificity of 0.76 (95% CI 0.67‐0.83) and 0.69 (95% CI 0.65‐0.73); the clinical feature+X-ray+MRI–based model had a pooled C-index of 0.818 (95% CI 0.709‐0.944; *z* score=−2.74; *P*=.006; Figure S6A in Multimedia Appendix 1).

In the aforementioned main meta-analysis, the PIs for all pooled C-indices were broad, with lower limits below 0.7 (C-index >0.7 suggests satisfactory discriminatory power of the model). Therefore, subgroup analyses were conducted (Figures S1-S6B in Multimedia Appendix 1). Subgroup analyses by the validation method revealed that the pooled C-index of X-ray–based and X-ray+clinical feature–based models was superior in independent validation to that in cross-validation, whereas the pooled C-index of clinical feature–, MRI-, and MRI+clinical feature–based models was superior in cross-validation to that in independent validation (Tables S3 and S4 in Multimedia Appendix 1).

#### Other Progression

Other KOA progression included pain progression, dysfunction progression, and stiffness progression. For predicting other progression, the clinical feature–based model had a pooled C-index of 0.746 (95% CI 0.680‐0.817; 95% PI: 0.532‐1.000; *z* score=−6.27; *P*<.001; Figure S7A in Multimedia Appendix 1), with sensitivity and specificity of 0.67 (95% CI 0.51‐0.80) and 0.80 (95% CI 0.72‐0.87); the MRI-based model had a pooled C-index of 0.799 (95% CI 0.746‐0.857; 95% PI 0.640‐0.998; *z* score=−6.33; *P*<.001; Figure S8A in Multimedia Appendix 1), with sensitivity and specificity of 0.74 (95% CI 0.66‐0.81) and 0.77 (95% CI 0.73‐0.80); the X-ray–based model had a pooled C-index of 0.687 (95% CI 0.550‐0.859; *z* score=−3.29; *P*=.001; Figure S9A in Multimedia Appendix 1); the MRI+clinical feature–based model had a pooled C-index of 0.820 (95% CI 0.773‐0.869; 95% PI 0.676‐0.994; *z* score=−6.70; *P*<.001; Figure S10A in Multimedia Appendix 1), with sensitivity and specificity of 0.77 (95% CI 0.72‐0.81) and 0.76 (95% CI 0.71‐0.80); the X-ray+clinical feature–based model had a pooled C-index of 0.788 (95% CI 0.735‐0.845; 95% PI 0.610‐1.000; *z* score=−6.72; *P*<.001; Figure S11A in Multimedia Appendix 1), with sensitivity and specificity of 0.68 (95% CI 0.65‐0.71) and 0.80 (95% CI 0.76‐0.83); the clinical feature+X-ray+MRI–based model had a pooled C-index of 0.712 (95% CI 0.645‐0.787; 95% PI 0.495‐1.000; *z* score=−6.69; *P*<.001; Figure S12A in Multimedia Appendix 1), with sensitivity and specificity of 0.66 (95% CI 0.54‐0.75) and 0.71 (95% CI 0.61‐0.80).

In the aforementioned main meta-analysis, the PIs for all pooled C-indices were broad, with lower limits below 0.7 (C-index >0.7 suggests satisfactory discriminatory power of the model). Therefore, subgroup analyses were conducted (Figures S7-S12B in Multimedia Appendix 1). Subgroup analyses by the validation method revealed that the pooled C-index of X-ray–based and X-ray+clinical feature–based models was superior in independent validation to that in cross-validation, whereas the pooled C-index of clinical feature–, MRI-, MRI+clinical feature–, or clinical feature+X-ray+MRI–based models was superior in cross-validation to that in independent validation (Tables S5 and S6 in Multimedia Appendix 1).

## Discussion

### Summary of the Main Findings

In this systematic review of the predictive value of ML in KOA progression, 32 observational studies were included. The predictive performance of ML, traditional methods, and DL was systematically compared under different validation methods, model types, modeling variables, and definitions of KOA progression. KOA progression in the included studies was defined primarily as imaging progression and other types of progression, including pain progression, dysfunction progression, and stiffness progression. The modeling variables mainly included traditional clinical features and imaging features (X-ray and MRI), as well as clinical feature+X-ray or MRI, and clinical feature+X-ray+MRI in a small number of studies. The C-index measures a prediction model’s discriminatory power, and a C-index >0.7 indicates satisfactory performance and practical value [52]. For predicting all progression in this systematic review, ML models demonstrated robust performance under different modeling variables, with pooled C-indices >0.7 and lower limits of PIs >0.5 in the validation cohort, suggesting that they possess clinically significant discriminatory power in predicting KOA progression. The clinical feature–based model was established mainly by logistic regression and exhibited accuracy comparable to other ML models. Among image-based models, traditional ML or DL possessed higher accuracy.

### Comparison With Previous Reviews

Castagno et al [12] described the application of ML in OA rather than in KOA, provided qualitative descriptions of algorithm type distributions and validation strategies, and broadly reviewed the application value of ML. However, they did not statistically compare the models’ actual efficacy, so their conclusions lacked quantitative evidence, restricting readers’ understanding of the ML predictive value in progression. Miraj et al [13] only described the ML model for predicting KOA progression, compared the accuracy of different ML models, and reported the original study results in descriptive tables. However, they conducted no lateral comparison of algorithms, and the pooled effect sizes were not calculated by meta-analyses, so their conclusion lacked high-grade evidence in evidence-based medicine. Ramazanian et al [11] conducted a qualitative overview of prediction models for KOA and listed the AUCs for traditional statistical models and ML models. However, they did not directly compare the predictive efficacy between the 2 models, and no pooled analyses were conducted on the models’ C-index, sensitivity, and specificity. This is the first systematic review and meta-analysis assessing the predictive value of ML in KOA progression under different model types, modeling variables, and definitions of KOA progression and revealing the application status of ML in predicting KOA progression.

### Definitions of KOA Progression

Despite the high accuracy of ML in predicting KOA progression, some challenges are still present. First, the definition of KOA progression was mainly based on dynamic changes in imaging and clinical symptoms, but it varied across the included studies. Generally, KOA progression is categorized into imaging and clinical symptom progression. Imaging progression is assessed using X-ray and has different definitions across studies, mainly including JSW reduction and an increase in KL grade. The JSW reduction is defined as ≥0.7 mm in most studies, ≥0.5 mm in the study by Schiratti et al [43], and ≥0.6 mm in the study by Castagno et al [24]. Moreover, errors may be produced in measurements of JSW due to variations in position or observer interpretation, affecting model accuracy. The different definitions of JSW reduction also restrict the generalizability of corresponding prediction models.

Besides, the definition of the increase in KL grade is also different, including 1 or greater during follow-up [48], 2 or greater [49], and any value in most studies. A few studies also defined imaging progression as loss of cartilage volume and an increase in the number of osteophytes [23,35]. In addition, clinical symptom progression, including pain, dysfunction, stiffness, and overall progression, is mostly assessed by the WOMAC scale (pain, stiffness, and physical function). Pain, dysfunction, and stiffness progression are defined as an increase in the score of the corresponding dimension, and overall progression is defined as an increase in the overall WOMAC score. For the same progression, however, the increase in the WOMAC score has different definitions, such as 9 or higher during follow-up in most studies, and 2 or higher in the study by Castagno et al [24]. To sum up, KOA progression has not been clearly defined, which may have some impact on the effectiveness of progression assessment tools or independent prediction tools.

### Comparison of Modeling Variables

Modeling variables are key contributors to the performance of ML models, so selecting appropriate variables is important. In this systematic review and meta-analysis, modeling variables mainly included traditional clinical features and imaging features (X-ray and MRI). In addition, the combination of different modeling variables was also used [24,35,40]. Traditional clinical features are suitable for establishing models with stronger interpretability, but they are often less accurate for predicting positive events, as reflected in the model performance in the validation cohort of this systematic review. Imaging-based models have attracted widespread attention recently, which are established essentially by segmenting medical images and then extracting and filtering features, such as texture structure, achieving satisfactory effects in many fields [53]. The imaging-based model exhibited high accuracy in both cross-validation and independent validation in this systematic review.

However, some challenges are present during image feature extraction. First, the different image quality may affect the imaging results, and MRI can capture more image features than X-ray. This systematic review also showed that the accuracy of the MRI-based model was higher than that of the X-ray–based model, but the clinical diagnosis and treatment of KOA are mainly dependent on X-ray at present, thus greatly hindering the generalizability or utility of the MRI-based model. Second, image segmentation is easily susceptible to prior knowledge or experience, which may result in some variations in the segmentation of regions of interest across studies, thus affecting the predictive effect. Finally, large amounts of information will be excluded during the screening of texture structure, also producing a risk of information loss. Genetic information–based models usually involve a small number of cases, so they are prone to overfitting or insufficient statistical power [54]. Besides, the combination of different modeling variables made the model more complex but did not enhance its accuracy in this systematic review, which may be related to the modeling variables and the data integration mode. In the future, the multiomics data integration model should be further optimized to raise the model’s predictive accuracy.

### Comparison of Model Types

The type of task, as well as the model accuracy and interpretability, must be taken into account when constructing a prediction model. In this systematic review and meta-analysis, logistic regression models and some of the traditional ML models (eg, decision trees and random forests) possessed excellent interpretability, but they tended to be less accurate, whereas models with poor interpretability (eg, support vector machine, neural network, and DL) were more accurate [55]. Therefore, both interpretability and accuracy should be considered when establishing models based on interpretable clinical features. In addition, DL models are superior to traditional ML models in image processing. The reason is that traditional ML requires image segmentation, and its texture feature extraction is highly susceptible to personal experience, whereas DL models can be directly established based on the image, with texture segmentation and extraction incorporated into training, thereby maximizing the retention of image information and improving the intelligent performance [56]. Due to the limited number of studies included and the single data extracted, however, the great advantage of DL was not verified in this systematic review. Therefore, DL-based image processing is pending further exploration in the future.

### Comparison of Validation Methods

In the included studies, the validation cohorts were generated by internal (cross-validation) or external validation. By dividing the dataset into multiple subsets followed by training and testing, cross-validation can avoid the random bias of a single division, allow for a more robust estimate of the generalizability, and also help optimize the parameters and avoid overfitting, thereby achieving a balance between efficient training and validation with limited data [57]. In this systematic review and meta-analysis, external validation with independent datasets was used in only 7 of the 30 included studies, and the models were generally less accurate in external validation than in the training cohort, indicating that overfitting may still be present in the training cohort despite cross-validation. To sum up, despite a high accuracy, ML models lack external and clinical validation in most studies, and their reliability and generalizability remain inconclusive in different clinical settings, hindering their utility in KOA management. In addition, the performance of ML models in predicting KOA progression is influenced by a variety of factors, including sample size, data quality, and source. Therefore, these influencing factors should be fully considered when establishing and validating ML models, and model optimization and validation should be achieved in appropriate ways.

### Challenges in Model Uncertainty

This systematic review reported both CIs and PIs for effect sizes, which more accurately reflected the uncertainty of model performance across different clinical settings. Unlike CIs in traditional systematic reviews, PIs emphasize the range of potential model performance in “a future similar study” or “another real-world clinical population” [58]. The PIs observed in this study were markedly broader than the CIs, with some lower limits approaching random levels. This reveals a fundamental challenge for current ML models in predicting KOA progression, namely, significant uncertainty of their generalizability across different clinical settings. This uncertainty was often obscured in previous literature that only reported the mean C-index [20]. Although some pooled statistical indicators indicate satisfactory mean discriminatory power of the model, the broad PIs suggest that the actual predictive performance of the model may fluctuate greatly in specific new patient cohorts or independent external validation sets. This nonrobustness arises not only from algorithm differences but also more fundamentally from the heterogeneity of data sources in available studies and the nonstandardized definition of “disease progression.” Therefore, future research should not only focus on indicators for higher accuracy but also enhance the model robustness to narrow PIs, thereby enabling translation of ML models from the laboratory to clinical precision medicine.

### Clinical Application

The effect sizes were pooled using a random effects model, and PIs were reported. However, the PIs were broad in the results of some pooled analyses, indicating substantial unexplained heterogeneity in the actual predictive performance. To investigate potential sources of heterogeneity, subgroup analyses were conducted by the model type and modeling variable [59]. However, broad PIs remained in some subgroups, indicating substantial unexplained heterogeneity despite stratifications. This underscores the complexity of predicting KOA progression and the potential influence of factors beyond those in this systematic review and meta-analysis. Therefore, clinical decisions should be made cautiously in specific contexts. As discussed earlier, the potential risk of inconsistency increased due to different definitions of KOA progression, modeling variables, model types, and validation methods, underscoring the need for rigorously standardized workflows in future clinical translation of ML models. Meanwhile, subgroup analyses were conducted by the dataset, model type, modeling variable, and definition of KOA progression, and no specific model type that demonstrated optimal predictive performance was found. In clinical practice, traditional ML or logistic regression models can be established to construct risk scoring tools with better interpretability. To predict the risk, image processing can be achieved by DL. Before clinical popularization and application, these models require prospective validation in a real-world setting.

### Advantages and Limitations

This systematic review and meta-analysis on ML models for predicting KOA progression offered an evidence-based basis for the development or use of intelligent tools. However, some limitations are worth noting. First, a limited number of studies were included, and subgroup analyses were conducted by the model type, modeling variable, and definition of KOA progression, but the influence of follow-up duration on the modeling results was not deeply explored. Second, the definition of KOA progression varied across studies, restricting the popularization and application of prediction models. Third, the validation cohort was generated mainly by internal validation, and most studies lacked external validation, which weakened the potential of ML in clinical translation. Fourth, the variations in ML methods under different modeling variables were not investigated due to the limited studies included. Fifth, the datasets used were mostly from public databases, which may produce bias in specific patient groups or limitations in data collection. Finally, a random effects model was used to pool the effect sizes. However, the pooled C-indices of models established using different variables (clinical features, X-ray, MRI, and multiomics) were broad, indicating significant uncertainty in current conclusions. This uncertainty may be increased due to fewer studies included in specific subgroups, but its main contributors remain the substantial variation in true effect sizes across clinical settings and populations. This suggests that besides those covered in the subgroup analyses, other factors, such as variations in image acquisition, may also contribute to the heterogeneity. In the future, more studies are expected to emerge with rapid advancements in this field, enriching data for subsequent systematic reviews and meta-analyses.

### Conclusions

This systematic review systematically compared for the first time the performance of ML in predicting KOA progression under different model types, modeling variables, and definitions of KOA progression. Currently available reviews are mostly qualitative descriptions of the predictive value of ML in KOA progression, while this systematic review, combining CIs with PIs, systematically quantified the predictive efficacy boundaries of ML models for KOA progression. On the basis of the mean effect across included studies, ML models demonstrate certain discriminatory power in predicting KOA progression. However, current evidence should be interpreted with caution due to significant heterogeneity, such as variations in the definition of KOA progression, modeling variables, and validation strategies, and high RoB, as well as uncertainty in effect distribution as revealed by the broad PI. Future research should standardize the definition of KOA progression, enhance methodological rigor, and conduct stringent external validation to improve model reliability and clinical applicability.

## Supplementary material

10.2196/80430 Multimedia Appendix 1 Search strategy, quality assessment, and results of subgroup analyses.

10.2196/80430 Checklist 1 PRISMA-DTA checklist.

## Abbreviations

C-index: concordance index
DL: deep learning
JSW: joint space width
KL: Kellgren-Lawrence
KOA: knee osteoarthritis
LR: logistic regression
ML: machine learning
MRI: magnetic resonance imaging
OA: osteoarthritis
PI: prediction interval
RF: random forest
RoB: risk of bias
WOMAC: Western Ontario and McMaster Universities Osteoarthritis Index
XGBoost: Extreme Gradient Boosting
