# Supplementary material for: Predictive Value of Machine Learning in Knee Osteoarthritis Progression: Systematic Review and Meta-Analysis
Source: J Med Internet Res. 2025 Dec 30;27:e80430. doi: 10.2196/80430 (PMC12753132; doi:10.2196/80430)
Supplement: Multimedia Appendix 1 [file jmir-v27-e80430-s001.doc]

## Multimedia Appendix

**Supplementary Figures**

**
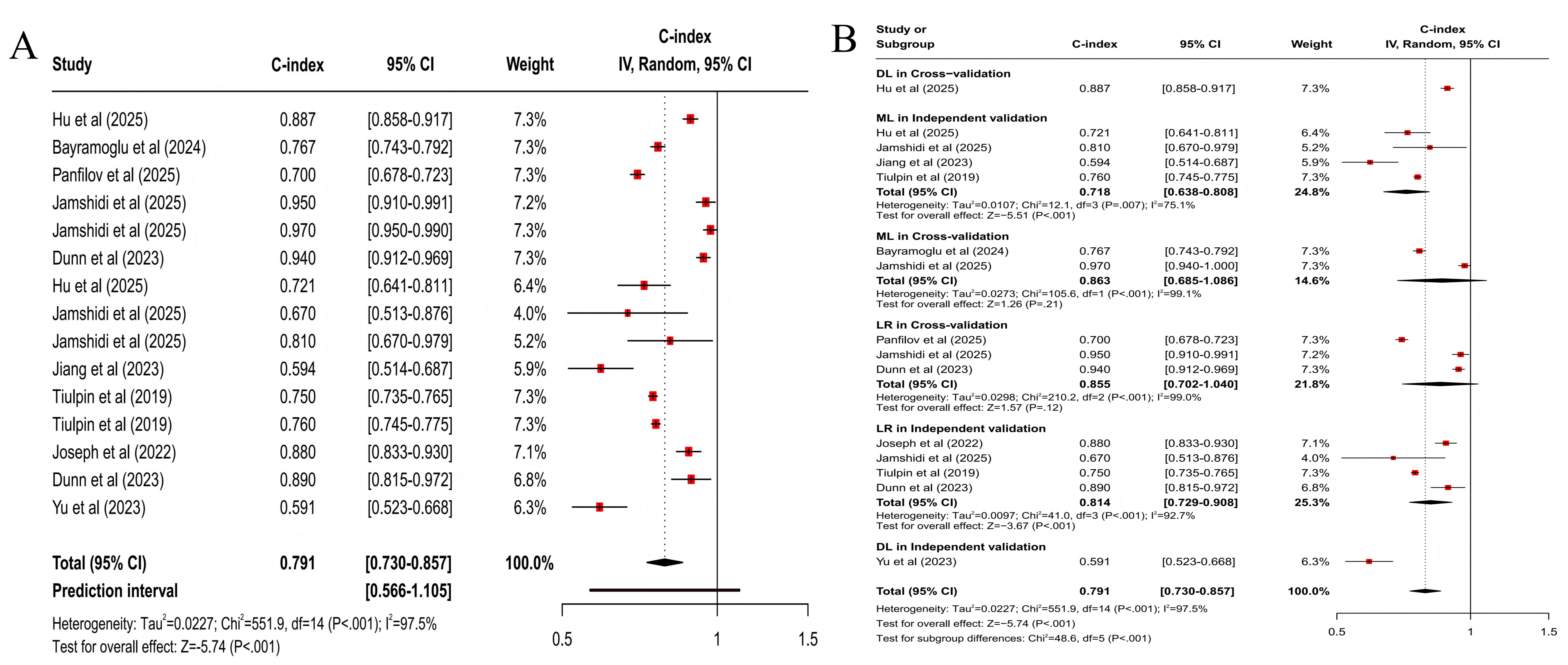
**

**Figure S1** Forest plot for meta-analysis of C-index of clinical feature-based model for predicting imaging progression of knee osteoarthritis. (A) Main meta-analysis; (B) subgroup analysis. DL: deep learning; LR: logistic regression; ML: machine learning.

**
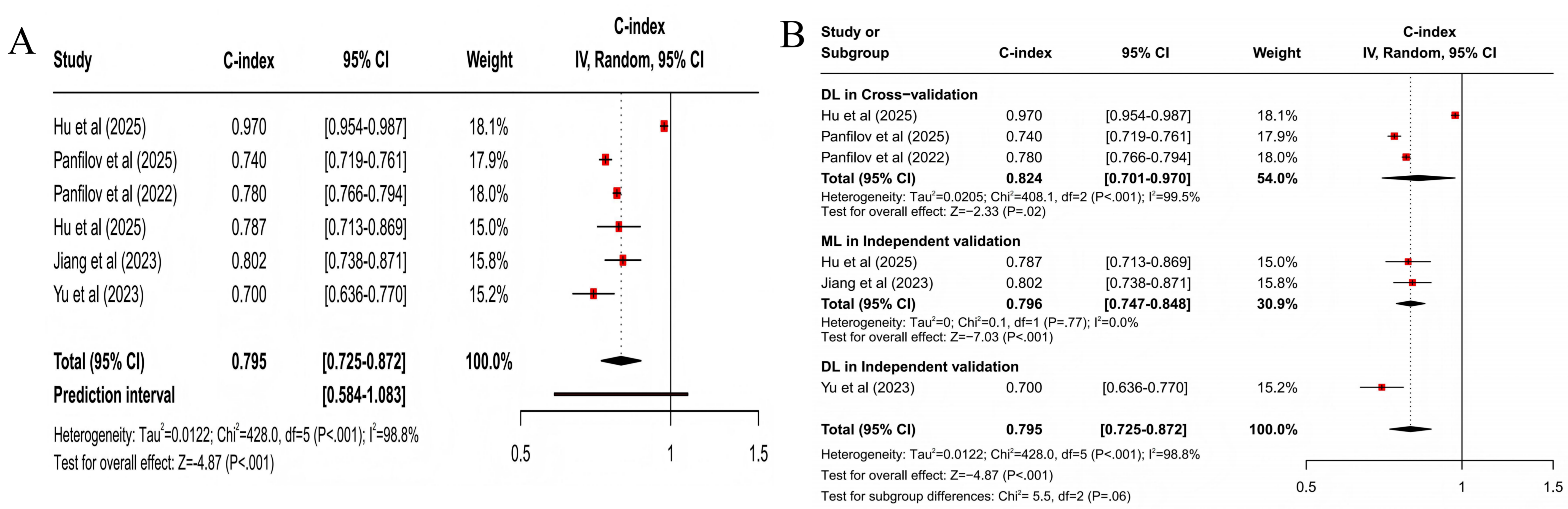
**

**Figure S2** Forest plot for meta-analysis of C-index of MRI-based model for predicting imaging progression of knee osteoarthritis. (A) Main meta-analysis; (B) subgroup analysis. DL: deep learning; LR: logistic regression; ML: machine learning.


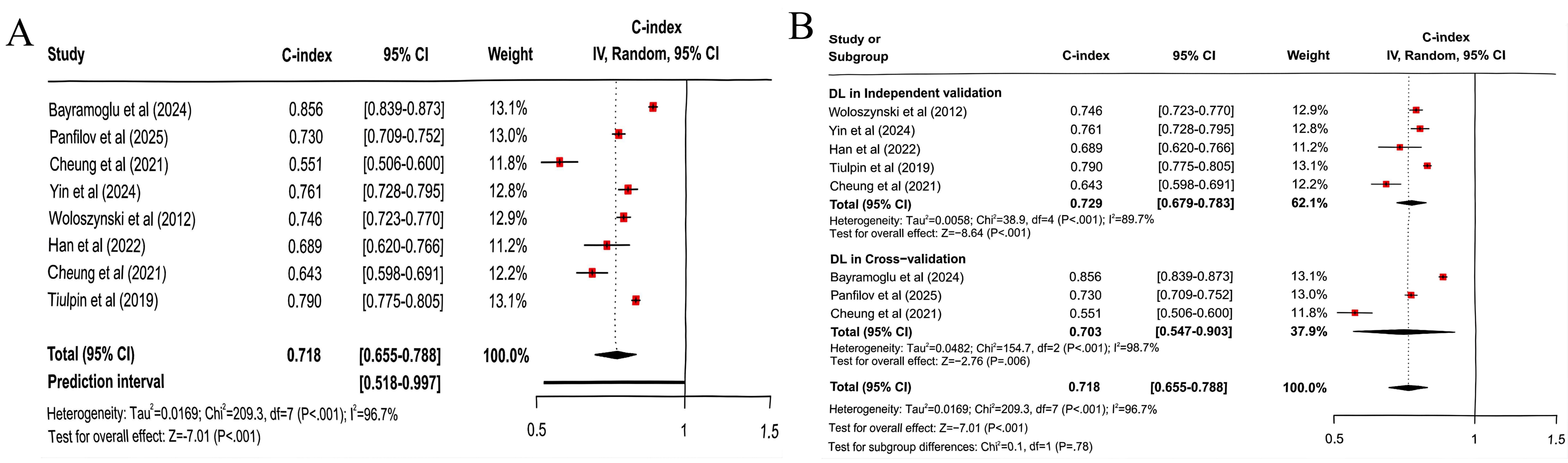


**Figure S3** Forest plot for meta-analysis of C-index of X-ray-based model for predicting imaging progression of knee osteoarthritis. (A) Main meta-analysis; (B) subgroup analysis. DL: deep learning; LR: logistic regression; ML: machine learning.


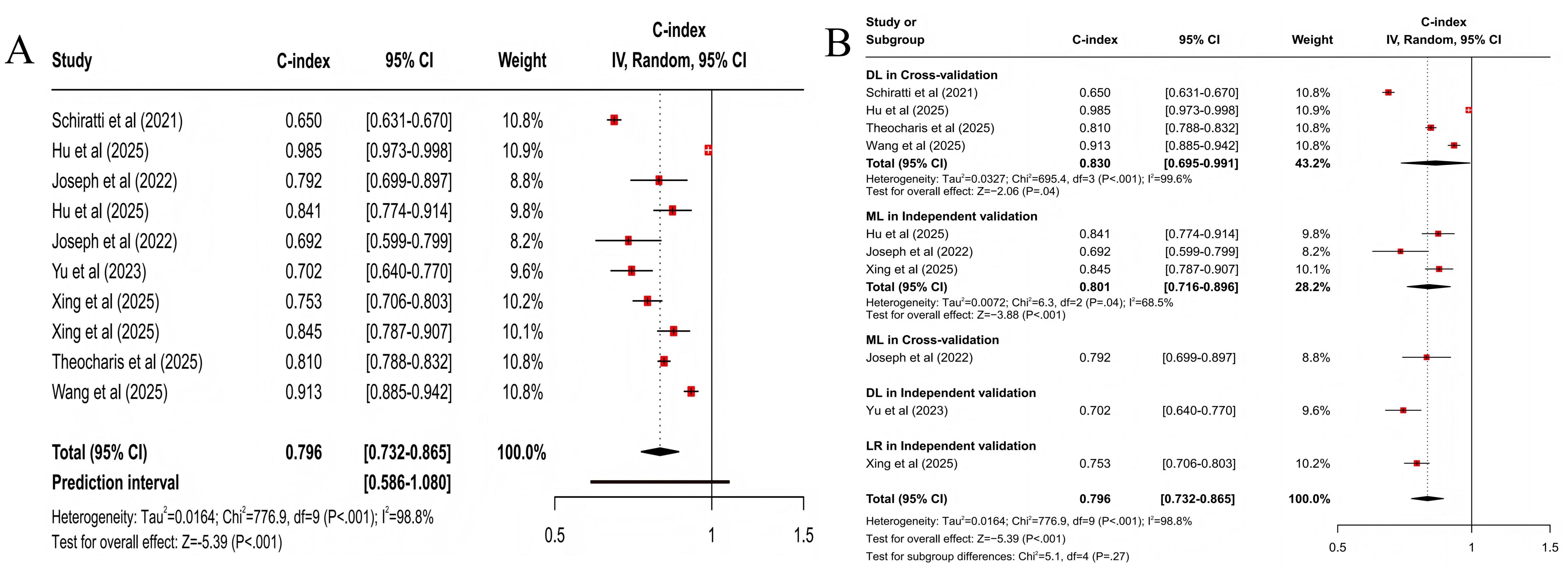


**Figure S4** Forest plot for meta-analysis of C-index of MRI + clinical feature-based model for predicting imaging progression of knee osteoarthritis. (A) Main meta-analysis; (B) subgroup analysis. DL: deep learning; LR: logistic regression; ML: machine learning.


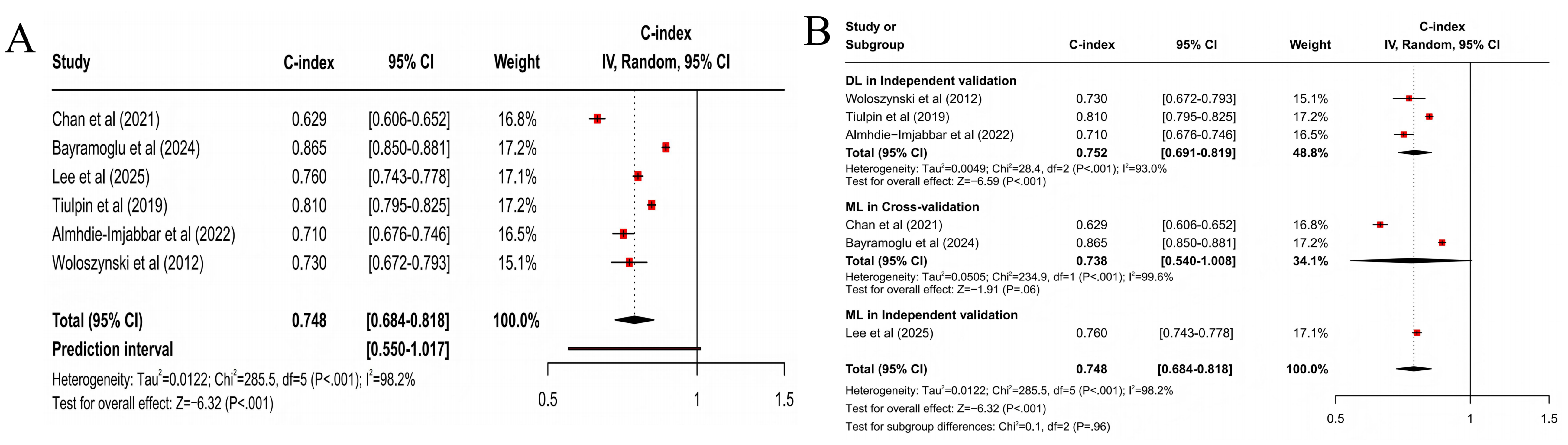


**Figure S5** Forest plot for meta-analysis of C-index of X-ray + clinical feature-based model for predicting imaging progression of knee osteoarthritis. (A) Main meta-analysis; (B) subgroup analysis. DL: deep learning; LR: logistic regression; ML: machine learning.


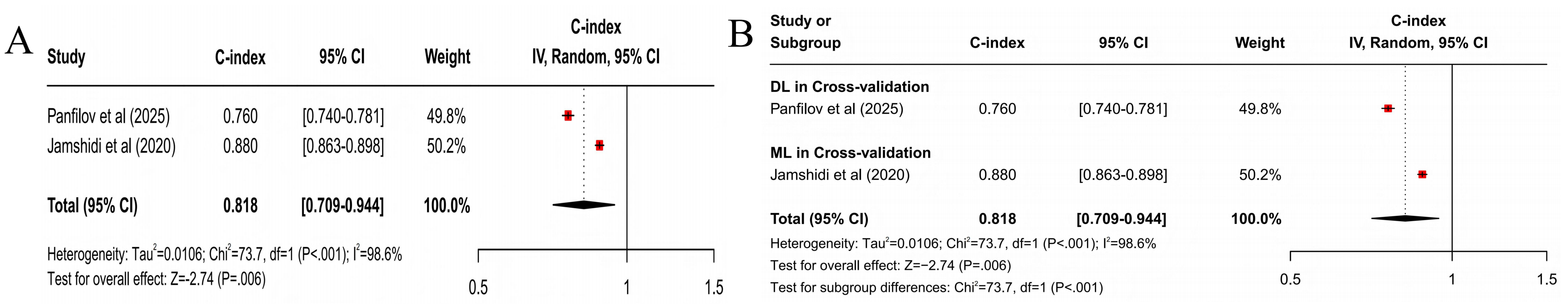


**Figure S6** Forest plot for meta-analysis of C-index of clinical feature + X-ray + MRI-based model for predicting imaging progression of knee osteoarthritis. (A) Main meta-analysis; (B) subgroup analysis. DL: deep learning; LR: logistic regression; ML: machine learning.


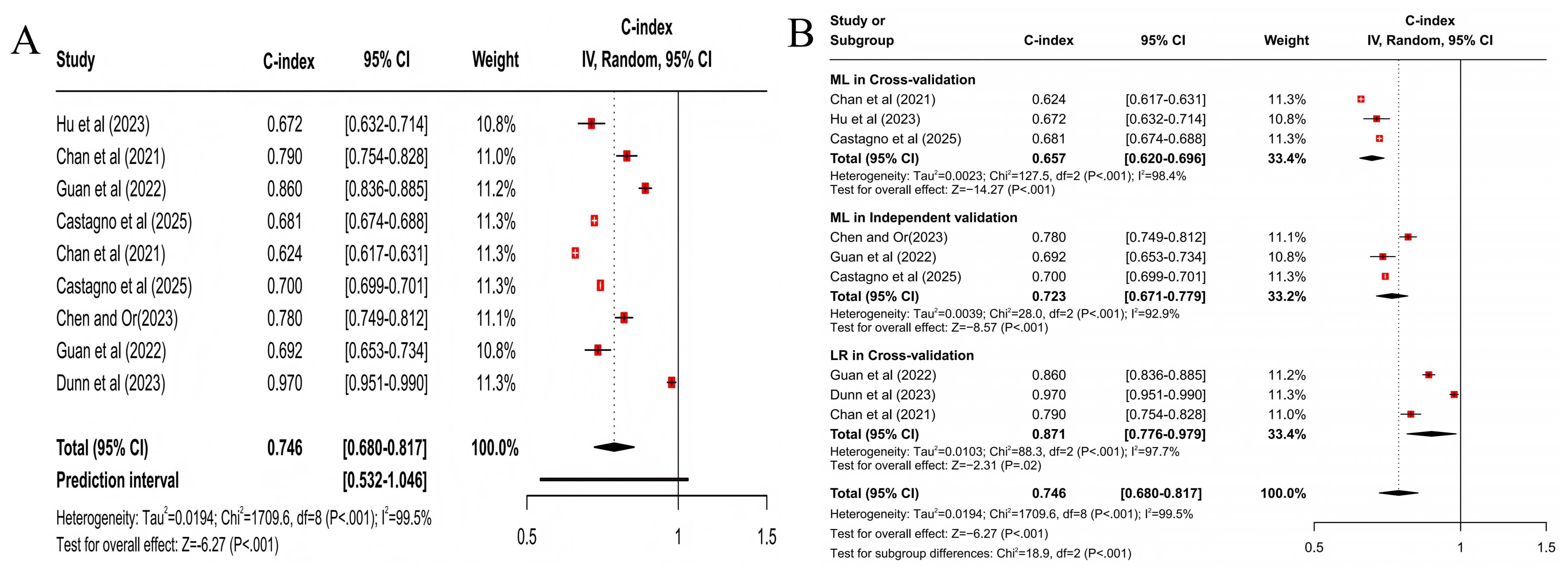


**Figure S7** Forest plot for meta-analysis of C-index of clinical feature-based model for predicting other progression of knee osteoarthritis. (A) Main meta-analysis; (B) subgroup analysis. DL: deep learning; LR: logistic regression; ML: machine learning.


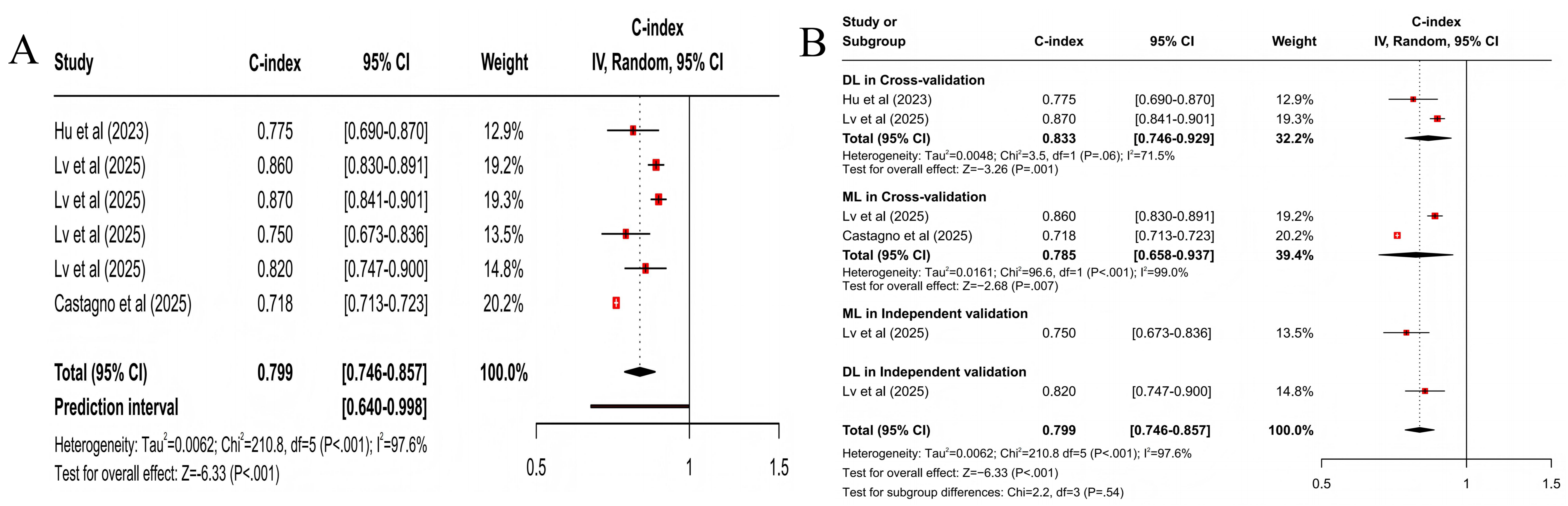


**Figure S8** Forest plot for meta-analysis of C-index of MRI-based model for predicting other progression of knee osteoarthritis. (A) Main meta-analysis; (B) subgroup analysis. DL: deep learning; LR: logistic regression; ML: machine learning.

**
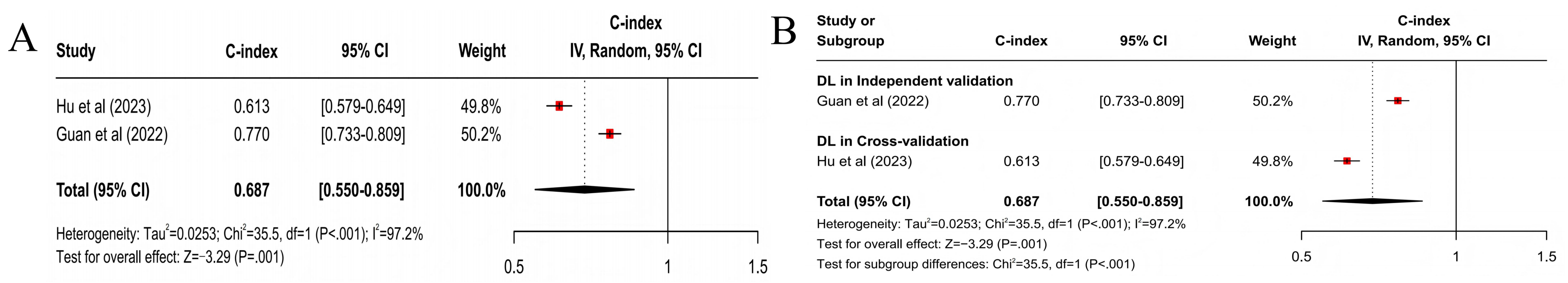
**

**Figure S9** Forest plot for meta-analysis of C-index of X-ray-based model for predicting other progression of knee osteoarthritis. (A) Main meta-analysis; (B) subgroup analysis. DL: deep learning; LR: logistic regression; ML: machine learning.


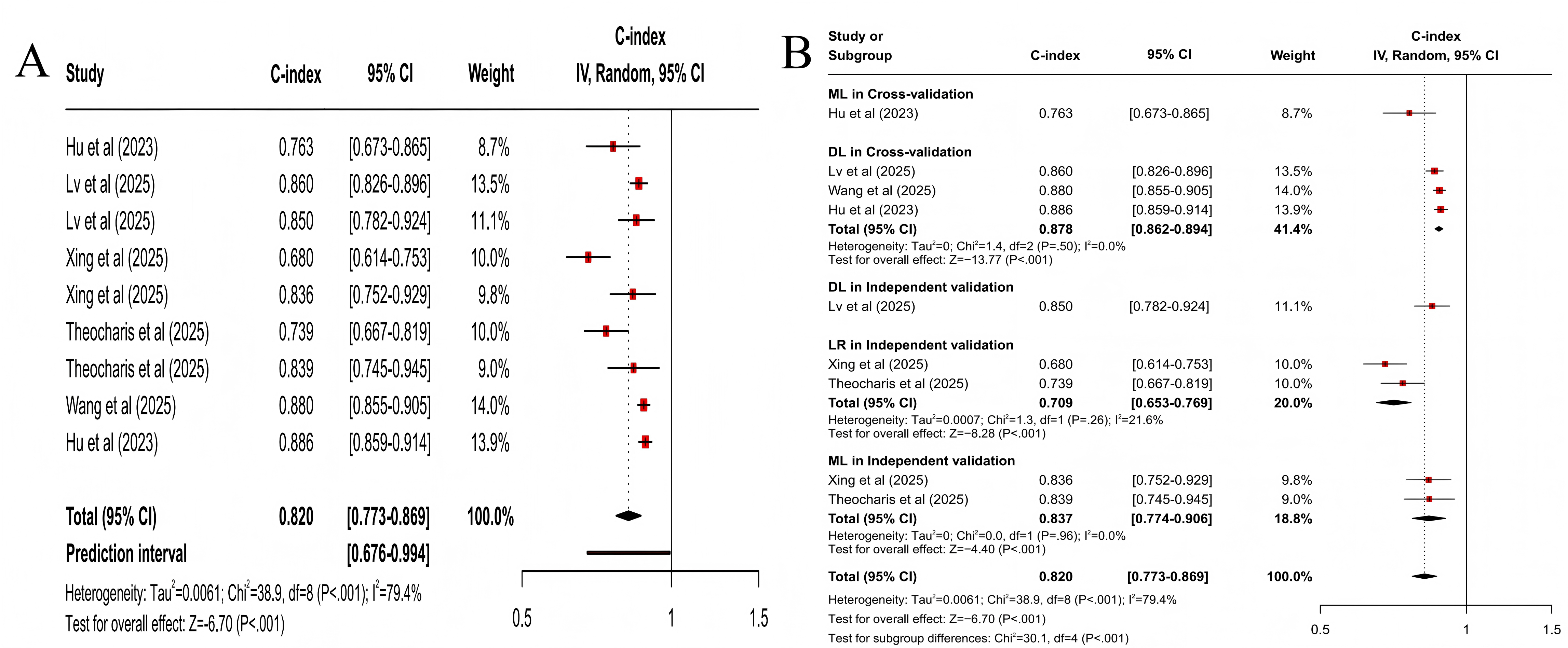


**Figure S10** Forest plot for meta-analysis of C-index of MRI + clinical feature-based model for predicting other progression of knee osteoarthritis. (A) Main meta-analysis; (B) subgroup analysis. DL: deep learning; LR: logistic regression; ML: machine learning.


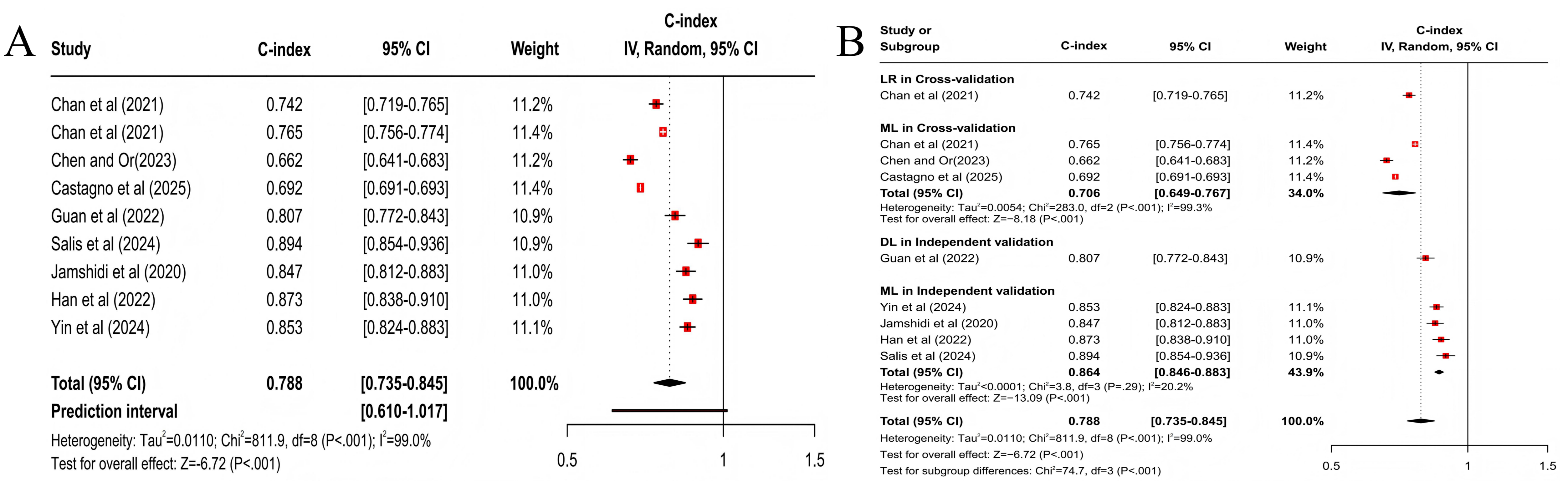


**Figure S11** Forest plot for meta-analysis of C-index of X-ray + clinical feature-based model for predicting other progression of knee osteoarthritis. (A) Main meta-analysis; (B) subgroup analysis. DL: deep learning; LR: logistic regression; ML: machine learning.


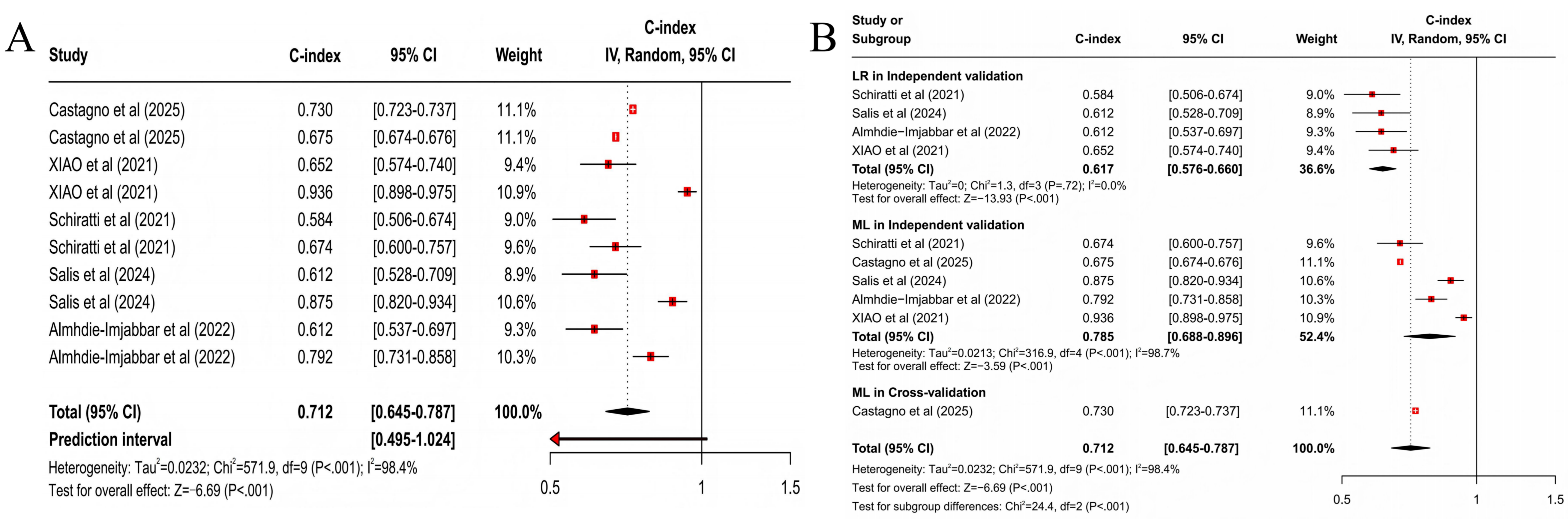


**Figure S12** Forest plot for meta-analysis of C-index of clinical feature + X-ray + MRI-based model for predicting other progression of knee osteoarthritis. (A) Main meta-analysis; (B) subgroup analysis. DL: deep learning; LR: logistic regression; ML: machine learning.

**Supplementary Tables**

**Table S1** Search strategy of this meta-analysis

**1.Pubmed**

| Search  number | Query | Results |
| --- | --- | --- |
| #1 | Osteoarthritis[MeSH Terms] | 86702 |
| #2 | "Arthroses"[Title/Abstract] OR "arthrosis"[Title/Abstract] OR "Degenerative  Arthritides"[Title/Abstract] OR "degenerative arthritis"[Title/Abstract] OR "degenerative joint disease"[Title/Abstract] OR "noninflammatory arthritis"[Title/Abstract] OR "osteo  arthritis"[Title/Abstract] OR "osteo arthrosis"[Title/Abstract] OR "Osteoarthritides"[Title/Abstract] OR "osteoarthritis"[Title/Abstract] OR "Osteoarthroses"[Title/Abstract] OR  "osteoarthrosis"[Title/Abstract] OR "Gonarthrosis"[Title/Abstract] | 116931 |
| #3 | Machine Learning[MeSH Terms] | 104441 |
| #4 | "learning machine*"[Title/Abstract] OR "machine learning"[Title/Abstract] OR "Transfer Learning"[Title/Abstract] OR "artificial intelligence"[Title/Abstract] OR "prediction  model"[Title/Abstract] OR "risk model"[Title/Abstract] OR "risk score"[Title/Abstract] OR "Deep learning"[Title/Abstract] OR "ResNet"[Title/Abstract] OR "AlexNet"[Title/Abstract] OR  "VGGNet"[Title/Abstract] OR "GoogLeNet"[Title/Abstract] OR "Ensemble  Learning"[Title/Abstract] OR "random forest"[Title/Abstract] OR "neural network"[Title/Abstract] OR "neural networks"[Title/Abstract] OR "CNN"[Title/Abstract] OR "K-Nearest  Neighbor"[Title/Abstract] OR "Support vector machine"[Title/Abstract] OR  "SVM"[Title/Abstract] OR "Gradient Boosting Machine"[Title/Abstract] OR  "Nomogram"[Title/Abstract] OR "XGBoost"[Title/Abstract] OR "Adaboost"[Title/Abstract] OR "LightGBM"[Title/Abstract] OR "CatBoost"[Title/Abstract] OR "Gradient  Boosting"[Title/Abstract] OR "Decision tree"[Title/Abstract] OR "Regression Trees"[Title/Abstract] OR "Naive Bayesian"[Title/Abstract] OR "Multilayer perceptron"[Title/Abstract] OR "Bayesian network"[Title/Abstract] OR  "Radiomics"[Title/Abstract] OR "Radiomic"[Title/Abstract] | 516611 |
| #5 | #1 OR #2 | 138141 |
| #6 | #3 OR #4 | 523970 |
| #7 | (#1 OR #2) AND (#3 OR #4) | 1831 |

**2.Cochrane**

| Search  number | Query | Results |
| --- | --- | --- |
| #1 | MeSH descriptor: [Osteoarthritis] explode all trees | 11440 |
| #2 | (Arthroses):ti,ab,kw OR (arthrosis):ti,ab,kw OR (Degenerative Arthritides):ti,ab,kw OR  (degenerative arthritis):ti,ab,kw OR (degenerative joint disease):ti,ab,kw | 1817 |
| #3 | (noninflammatory arthritis):ti,ab,kw OR (osteo arthritis):ti,ab,kw OR (osteo arthrosis):ti,ab,kw OR  (Osteoarthritides):ti,ab,kw OR (osteoarthritis):ti,ab,kw | 27009 |
| #4 | (Osteoarthroses):ti,ab,kw OR (osteoarthrosis):ti,ab,kw OR (Gonarthrosis):ti,ab,kw | 1111 |

| #5 | #1 or #2 or #3 or #4 | 27900 |
| --- | --- | --- |
| #6 | MeSH descriptor: [Machine Learning] explode all trees | 1211 |
| #7 | (learning machine*):ti,ab,kw OR (machine learning):ti,ab,kw OR (Transfer Learning):ti,ab,kw OR (artificial intelligence):ti,ab,kw OR (prediction model):ti,ab,kw | 14006 |
| #8 | (risk model):ti,ab,kw OR (risk score):ti,ab,kw OR (Deep learning):ti,ab,kw OR (ResNet):ti,ab,kw OR (AlexNet):ti,ab,kw | 74438 |
| #9 | (VGGNet):ti,ab,kw OR (GoogLeNet):ti,ab,kw OR (Ensemble Learning):ti,ab,kw OR (random  forest):ti,ab,kw OR (neural network):ti,ab,kw | 4218 |
| #10 | (neural networks):ti,ab,kw OR (CNN):ti,ab,kw OR (K-Nearest Neighbor):ti,ab,kw OR (Support  vector machine):ti,ab,kw OR (SVM):ti,ab,kw | 2949 |
| #11 | (Gradient Boosting Machine):ti,ab,kw OR (Nomogram):ti,ab,kw OR (XGBoost):ti,ab,kw OR  (Adaboost):ti,ab,kw OR (LightGBM):ti,ab,kw | 2258 |
| #12 | (CatBoost):ti,ab,kw OR (Gradient Boosting):ti,ab,kw OR (Decision tree):ti,ab,kw OR (Regression  Trees):ti,ab,kw OR (Naive Bayesian):ti,ab,kw | 1507 |
| #13 | (Multilayer perceptron):ti,ab,kw OR (Bayesian network):ti,ab,kw OR (Radiomics):ti,ab,kw OR (Radiomic):ti,ab,kw | 1314 |
| #14 | #6 or #7 or #8 or #9 or #10 or #11 or #12 or #13 | 89184 |
| #15 | #5 and #14 | 1213 |

**3.Embase**

| Search  number | Query | Results |
| --- | --- | --- |
| #1 | 'osteoarthritis'/exp | 197253 |
| #2 | 'arthroses':ab,ti OR 'arthrosis':ab,ti OR 'degenerative arthritides':ab,ti OR 'degenerative  arthritis':ab,ti OR 'degenerative joint disease':ab,ti OR 'noninflammatory arthritis':ab,ti OR 'osteo arthritis':ab,ti OR 'osteo arthrosis':ab,ti OR 'osteoarthritides':ab,ti OR 'osteoarthritis':ab,ti OR  'osteoarthroses':ab,ti OR 'osteoarthrosis':ab,ti OR 'gonarthrosis':ab,ti | 163608 |
| #3 | 'machine learning'/exp | 645838 |
| #4 | 'learning machine*':ab,ti OR 'machine learning':ab,ti OR 'transfer learning':ab,ti OR 'artificial  intelligence':ab,ti OR 'prediction model':ab,ti OR 'risk model':ab,ti OR 'risk score':ab,ti OR 'deep learning':ab,ti OR 'resnet':ab,ti OR 'alexnet':ab,ti OR 'vggnet':ab,ti OR 'googlenet':ab,ti OR  'ensemble learning':ab,ti OR 'random forest':ab,ti OR 'neural network':ab,ti OR 'neural  networks':ab,ti OR 'cnn':ab,ti OR 'k-nearest neighbor':ab,ti OR 'support vector machine':ab,ti OR 'svm':ab,ti OR 'gradient boosting machine':ab,ti OR 'nomogram':ab,ti OR 'xgboost':ab,ti OR  'adaboost':ab,ti OR 'lightgbm':ab,ti OR 'catboost':ab,ti OR 'gradient boosting':ab,ti OR 'decision  tree':ab,ti OR 'regression trees':ab,ti OR 'naive bayesian':ab,ti OR 'multilayer perceptron':ab,ti OR 'bayesian network':ab,ti OR 'radiomics':ab,ti OR 'radiomic':ab,ti | 604588 |
| #5 | #1 OR #2 | 229700 |
| #6 | #3 OR #4 | 905159 |
| #7 | #5 AND #6 | 4020 |

**4.Web of science**

| Search  number | Query | Results |
| --- | --- | --- |
| #1 | TS=("learning machine" OR "machine learning" OR "Transfer Learning" OR "artificial  intelligence" OR "prediction model" OR "risk model" OR "risk score" OR "Deep learning" OR  ResNet OR AlexNet OR VGGNet OR GoogLeNet OR "Ensemble Learning" OR "random forest" OR "neural network" OR "neural networks" OR CNN OR "K-Nearest Neighbor" OR "Support  vector machine" OR SVM OR "Gradient Boosting Machine" OR Nomogram OR XGBoost OR Adaboost OR LightGBM OR CatBoost OR "Gradient Boosting" OR "Decision tree" OR  "Regression Trees" OR "Naive Bayesian" OR "Multilayer perceptron" OR "Bayesian network " OR Radiomics OR Radiomic) | 1932966 |
| #2 | TS=(Arthroses OR arthrosis OR "Degenerative Arthritides" OR "degenerative arthritis" OR "degenerative joint disease" OR "noninflammatory arthritis" OR "osteo arthritis" OR "osteo arthrosis" OR Osteoarthritides OR osteoarthritis OR Osteoarthroses OR osteoarthrosis OR Gonarthrosis) | 151189 |
| #3 | #1 AND #2 | 2567 |

**Table S2** Risk of bias assessment results by PROBAST for included original studies

| No | Author | Year | Participants | | Predictors | | | Outcome | | | | | | Analysis | | | | | | | | |
| --- | --- | --- | --- | --- | --- | --- | --- | --- | --- | --- | --- | --- | --- | --- | --- | --- | --- | --- | --- | --- | --- | --- |
|  |  |  | Q1 | Q2 | Q1 | Q2 | Q3 | Q1 | Q2 | Q3 | Q4 | Q5 | Q6 | Q1 | Q2 | Q3 | Q4 | Q5 | Q6 | Q7 | Q8 | Q9 |
| 1 | Schiratti et al | 2021 | + | + | + | + | + | + | + | + | + | + | + | ? | + | + | + | + | + | + | ? | + |
| 2 | Woloszynski et al | 2012 | + | + | + | + | + | + | + | + | + | ? | + | - | + | + | + | + | + | + | ? | + |
| 3 | Chan et al | 2021 | + | + | + | + | + | + | + | + | + | + | + | ? | + | + | + | + | + | + | + | + |
| 4 | Du et al | 2018 | + | + | + | + | + | + | + | + | + | + | + | - | + | + | - | + | + | + | ? | + |
| 5 | Hu et al | 2025 | + | + | + | + | + | + | + | + | + | + | + | + | + | + | + | + | + | + | + | + |
| 6 | Chen et al | 2023 | + | + | + | + | + | + | + | + | + | + | + | ? | + | + | + | + | + | + | + | + |
| 7 | Guan et al | 2022 | + | + | + | + | + | + | + | + | + | + | + | + | + | + | + | + | + | + | ? | + |
| 8 | Bayramoglu et al | 2024 | + | + | + | + | + | + | + | + | + | + | + | + | + | + | - | + | + | + | + | + |
| 9 | Hu et al | 2023 | + | + | + | + | + | + | + | + | + | + | + | ? | + | + | + | + | + | + | + | + |
| 10 | Lee et al | 2025 | + | + | + | + | + | + | + | + | + | + | + | ? | + | + | - | + | + | + | ? | + |
| 11 | Panfilov et al | 2025 | + | + | + | + | + | + | + | + | + | + | + | + | + | + | + | + | + | + | + | + |
| 12 | Yin et al | 2024 | + | + | + | + | + | + | + | + | + | + | + | ? | + | + | + | + | + | + | ? | + |
| 13 | Jamshidi et al | 2020 | + | + | + | + | + | + | + | + | + | + | + | ? | + | + | + | + | + | + | + | + |
| 14 | Han et al | 2022 | + | + | + | + | + | + | + | + | + | + | + | ? | + | + | + | + | + | + | ? | + |
| 15 | Lv et al | 2025 | + | + | + | + | + | + | + | + | + | + | + | ? | + | + | - | + | + | + | ? | + |
| 16 | Joseph et al | 2022 | + | + | + | + | + | + | + | + | + | + | + | ? | + | + | - | + | + | + | + | + |
| 17 | Jamshidi et al | 2025 | + | + | + | + | + | + | + | + | + | + | + | + | + | + | + | + | + | + | + | + |
| 18 | Jiang et al | 2023 | + | + | + | + | + | + | + | + | + | + | + | + | + | + | + | + | + | + | + | + |
| 19 | Tiulpin et al | 2019 | + | + | + | + | + | + | + | + | + | + | + | + | + | - | + | + | + | + | + | + |
| 20 | Dunn et al | 2023 | + | + | + | + | + | + | + | + | + | + | + | - | + | + | - | + | + | + | ? | + |
| 21 | Ashinsky et al | 2017 | + | + | + | + | + | + | + | + | + | + | + | - | + | + | + | + | + | + | ? | + |
| 22 | Panfilov et al | 2022 | + | + | + | + | + | + | + | + | + | + | + | ? | + | + | + | + | + | + | ? | + |
| 23 | Castagno et al | 2025 | + | + | + | + | + | + | + | + | + | + | + | + | + | + | + | + | + | + | + | + |
| 24 | Salis et al | 2024 | + | + | + | + | + | + | + | + | + | + | + | + | + | + | - | + | + | + | ? | + |
| 25 | Yu et al | 2023 | + | + | + | + | + | + | + | + | + | + | + | + | + | + | + | + | + | + | + | + |
| 26 | Almhdie-Imjabbar et al | 2022 | + | + | + | + | + | + | + | + | + | + | + | + | + | + | + | + | + | + | + | + |
| 27 | XIAO et al | 2021 | + | + | + | + | + | + | + | + | + | + | + | ? | + | + | + | + | + | + | ? | + |
| 28 | Xing et al | 2025 | + | + | + | + | + | + | + | + | + | + | + | ? | + | + | + | + | + | + | ? | + |
| 29 | Cheung et al | 2021 | + | + | + | + | + | + | + | + | + | + | + | ? | + | + | - | + | + | + | + | + |
| 30 | Du et al | 2018 | + | + | + | + | + | + | + | + | + | + | + | - | + | + | - | + | + | + | + | + |
| 31 | Theocharis et al | 2025 | + | + | + | + | + | + | + | + | + | + | + | - | + | + | - | + | + | + | ? | + |
| 32 | Wang et al | 2025 | + | + | + | + | + | + | + | + | + | + | + | + | + | + | + | + | + | + | + | + |

Note: Q: question; +: low risk of bias; -: high risk of bias; ?: unclear.

**Table S3** Results of meta-analysis of C-index of ML for predicting imaging progression of KOA

| Modeling variables | Model | Cross-validation | | | | | Independent validation | | | | | Overall | | | | |
| --- | --- | --- | --- | --- | --- | --- | --- | --- | --- | --- | --- | --- | --- | --- | --- | --- |
|  |  | n | c-index  (95%CI) | PI | τ | τ² | n | c-index  (95%CI) | PI | τ | τ² | n | c-index  (95%CI) | PI | τ | τ² |
| Clinical features |  |  |  |  |  |  |  |  |  |  |  |  |  |  |  |  |
|  | LR | 3 | 0.855  (0.702-1.000) |  | 0.1726 | 0.0298 | 4 | 0.814  (0.729-0.908) |  | 0.0987 | 0.0097 | 7 | 0.830  (0.751-0.918) | 0.592-1.000 | 0.1282 | 0.0164 |
|  | ML | 2 | 0.863  (0.685-1.000) |  | 0.1652 | 0.0273 | 4 | 0.718  (0.638-0.808) |  | 0.1032 | 0.0107 | 6 | 0.767  (0.675-0.872) | 0.503-1.000 | 0.1505 | 0.0227 |
|  | DL | 1 | 0.887  (0.858-0.917) |  |  |  | 1 | 0.591  (0.519-0.663) |  |  |  | 2 | 0.727  (0.489-1.000) |  | 0.2834 | 0.0803 |
|  | Overall | 6 | 0.863  (0.775-0.960) | 0.597-1.000 | 0.1322 | 0.0175 | 9 | 0.740  (0.670-0.818) | 0.527-1.000 | 0.1386 | 0.0192 | 15 | 0.791  (0.730-0.857) | 0.566-1.000 | 0.1507 | 0.0227 |
| MRI features |  |  |  |  |  |  |  |  |  |  |  |  |  |  |  |  |
|  | LR |  |  |  |  |  |  |  |  |  |  |  |  |  |  |  |
|  | ML |  |  |  |  |  | 2 | 0.796  (0.747-0.848) |  | 0 | 0 | 2 | 0.796  (0.747-0.848) |  | 0 | 0 |
|  | DL | 3 | 0.824  (0.701-0.970) |  | 0.1431 | 0.0205 | 1 | 0.700  (0.636-0.770) |  |  |  | 4 | 0.794  (0.690-0.914) | 0.480-1.000 | 0.1409 | 0.0198 |
|  | Overall | 3 | 0.824  (0.701-0.970) | 0.405-1.000 | 0.1431 | 0.0205 | 3 | 0.763  (0.702-0.829) | 0.563-1.000 | 0.0564 | 0.0032 | 6 | 0.795  (0.725-0.872) | 0.584-1.000 | 0.1104 | 0.0122 |
| X-Ray features |  |  |  |  |  |  |  |  |  |  |  |  |  |  |  |  |
|  | LR |  |  |  |  |  |  |  |  |  |  |  |  |  |  |  |
|  | ML |  |  |  |  |  |  |  |  |  |  |  |  |  |  |  |
|  | DL | 3 | 0.703  (0.547-0.903) |  | 0.2195 | 0.0482 | 5 | 0.729  (0.679-0.783) |  | 0.0761 | 0.0058 | 8 | 0.719  (0.655-0.788) | 0.518-0.997 | 0.1301 | 0.0169 |
|  | Overall | 3 | 0.703  (0.547-0.903) | 0.236-1.000 | 0.2195 | 0.0482 | 5 | 0.729  (0.679-0.783) | 0.577-0.922 | 0.0761 | 0.0058 | 8 | 0.718  (0.655-0.788) | 0.518-0.997 | 0.1301 | 0.0169 |
| MRI + Clinical features |  |  |  |  |  |  |  |  |  |  |  |  |  |  |  |  |
|  | LR |  |  |  |  |  | 1 | 0.753  (0.706-0.803) |  |  |  | 1 | 0.753  (0.705-0.801) |  |  |  |
|  | ML | 1 | 0.792  (0.699-0.897) |  |  |  | 3 | 0.801  (0.716-0.896) |  | 0.0851 | 0.0072 | 4 | 0.804  (0.745-0.868) | 0.643-1.000 | 0.0583 | 0.0034 |
|  | DL | 4 | 0.830  (0.695-0.991) |  | 0.1809 | 0.0327 | 1 | 0.702  (0.640-0.770) |  |  |  | 5 | 0.804  (0.690-0.937) | 0.474-1.000 | 0.1730 | 0.0299 |
|  | Overall | 5 | 0.823  (0.714-0.949) | 0.505-1.000 | 0.1598 | 0.0255 | 5 | 0.770  (0.710-0.835) | 0.600-0.989 | 0.0801 | 0.0064 | 10 | 0.796  (0.732-0.865) | 0.586-1.000 | 0.1281 | 0.0164 |
| X-Ray + Clinical features |  |  |  |  |  |  |  |  |  |  |  |  |  |  |  |  |
|  | LR |  |  |  |  |  |  |  |  |  |  |  |  |  |  |  |
|  | ML | 2 | 0.738  (0.540-1.000) |  | 0.2248 | 0.0505 | 1 | 0.760  (0.743-0.778) |  |  |  | 3 | 0.745  (0.622-0.893) | 0.337-1.000 | 0.1594 | 0.0254 |
|  | DL |  |  |  |  |  | 3 | 0.752  (0.691-0.819) |  | 0.0698 | 0.0049 | 3 | 0.752  (0.691-0.819) | 0.528-1.000 | 0.0698 | 0.0049 |
|  | Overall | 2 | 0.738  (0.540-1.000) |  | 0.2248 | 0.0505 | 4 | 0.756  (0.712-0.801) | 0.618-0.923 | 0.0554 | 0.0031 | 6 | 0.748  (0.684-0.818) | 0.550-1.000 | 0.1104 | 0.0122 |
| X-Ray + MRI + Clinical features |  |  |  |  |  |  |  |  |  |  |  |  |  |  |  |  |
|  | LR |  |  |  |  |  |  |  |  |  |  |  |  |  |  |  |
|  | ML | 1 | 0.880  (0.862-0.898) |  |  |  |  |  |  |  |  | 1 | 0.880  (0.863-0.898) |  |  |  |
|  | DL | 1 | 0.760  (0.740-0.780) |  |  |  |  |  |  |  |  | 1 | 0.760  (0.740-0.781) |  |  |  |
|  | Overall | 2 | 0.818  (0.709-0.944) |  | 0.1030 | 0.0106 |  |  |  |  |  | 2 | 0.818  (0.709-0.944) |  | 0.1030 | 0.0106 |

Note: n: number of models; PI: prediction interval; LR: logistic regression; ML:machine learning; DL: deep learning.

**Table S4** Results of meta-analysis of sensitivity and specificity of ML for predicting imaging progression of KOA

| Modeling variables | Model | Cross-validation | | | Independent validation | | | Overall | | |
| --- | --- | --- | --- | --- | --- | --- | --- | --- | --- | --- |
|  |  | n | SE(95%CI) | SP(95%CI) | n | SE(95%CI) | SP(95%CI) | n | SE(95%CI) | SP(95%CI) |
| Clinical features |  |  |  |  |  |  |  |  |  |  |
|  | LR | 2 | 0.88-0.96 | 0.58-0.88 | 4 | 0.72(0.65-0.79) | 0.78(0.67-0.87) | 6 | 0.85(0.58-0.96) | 0.79(0.69-0.87) |
|  | ML | 2 | 0.70-0.95 | 0.72-0.83 | 4 | 0.79(0.70-0.86) | 0.55(0.29-0.78) | 6 | 0.81(0.71-0.89) | 0.64(0.43-0.80) |
|  | DL | 1 | 0.80 | 0.84 | 1 | 0.64 | 0.42 | 2 | 0.64-0.80 | 0.42-0.84 |
|  | Overall | 5 | 0.92(0.72-0.98) | 0.79(0.68-0.86) | 9 | 0.73(0.66-0.79) | 0.66(0.49-0.79) | 14 | 0.81(0.72-0.88) | 0.71(0.60-0.80) |
| MRI features |  |  |  |  |  |  |  |  |  |  |
|  | LR |  |  |  |  |  |  |  |  |  |
|  | ML |  |  |  | 2 | 0.77-0.83 | 0.68-0.80 | 2 | 0.77-0.83 | 0.68-0.80 |
|  | DL | 2 | 0.68-0.91 | 0.75-0.85 | 1 | 0.53 | 0.80 | 3 | 0.53-0.91 | 0.75-0.85 |
|  | Overall | 2 | 0.68-0.91 | 0.75-0.85 | 3 | 0.53-0.83 | 0.68-0.80 | 5 | 0.76(0.62-0.86) | 0.78(0.72-0.83) |
| X-Ray features |  |  |  |  |  |  |  |  |  |  |
|  | LR |  |  |  |  |  |  |  |  |  |
|  | ML |  |  |  |  |  |  |  |  |  |
|  | DL | 1 | 0.79 | 0.76 | 5 | 0.69(0.63-0.74) | 0.68(0.61-0.74) | 6 | 0.71(0.65-0.76) | 0.69(0.63-0.75) |
|  | Overall | 1 | 0.79 | 0.76 | 5 | 0.69(0.63-0.74) | 0.68(0.61-0.74) | 6 | 0.71(0.65-0.76) | 0.69(0.63-0.75) |
| MRI + Clinical features |  |  |  |  |  |  |  |  |  |  |
|  | LR |  |  |  | 1 | 0.81 | 0.60 | 1 | 0.81 | 0.60 |
|  | ML | 1 | 0.37 | 0.90 | 3 | 0.45-0.85 | 0.69-0.87 | 4 | 0.66(0.41-0.84) | 0.82(0.73-0.89) |
|  | DL | 3 | 0.84-0.95 | 0.41-0.93 | 1 | 0.64 | 0.70 | 4 | 0.85(0.71-0.93) | 0.77(0.53-0.91) |
|  | Overall | 4 | 0.80(0.54-0.93) | 0.83(0.60-0.94) | 5 | 0.73(0.58-0.83) | 0.73(0.64-0.81) | 9 | 0.77(0.63-0.87) | 0.78(0.66-0.86) |
| X-Ray + Clinical features |  |  |  |  |  |  |  |  |  |  |
|  | LR |  |  |  |  |  |  |  |  |  |
|  | ML | 2 | 0.62-0.85 | 0.66-0.75 | 1 | 0.77 | 0.65 | 3 | 0.62-0.85 | 0.65-0.75 |
|  | DL |  |  |  | 1 | 0.75 | 0.72 | 1 | 0.75 | 0.72 |
|  | Overall | 2 | 0.62-0.85 | 0.66-0.75 | 2 | 0.75-0.77 | 0.65-0.72 | 4 | 0.76(0.67-0.83) | 0.69(0.65-0.73) |
| X-Ray + MRI + Clinical features |  |  |  |  |  |  |  |  |  |  |
|  | LR |  |  |  |  |  |  |  |  |  |
|  | ML | 1 | 0.87 | 0.90 |  |  |  | 1 | 0.87 | 0.90 |
|  | DL |  |  |  |  |  |  |  |  |  |
|  | Overall | 1 | 0.87 | 0.90 |  |  |  | 1 | 0.87 | 0.90 |

Note: n: number of models; LR: logistic regression; ML:machine learning; DL: deep learning; SE: sensitivity; SP: specificity.

**Table S5** Results of meta-analysis of C-index of ML for predicting other progression of KOA

| Modeling variables | Model | Cross-validation | | | | | Independent validation | | | | | Overall | | | | |
| --- | --- | --- | --- | --- | --- | --- | --- | --- | --- | --- | --- | --- | --- | --- | --- | --- |
|  |  | n | c-index  (95%CI) | PI | τ | τ² | n | c-index  (95%CI) | PI | τ | τ² | n | c-index  (95%CI) | PI | τ | τ² |
| Clinical features |  |  |  |  |  |  |  |  |  |  |  |  |  |  |  |  |
|  | LR | 3 | 0.871  (0.776-0.979) |  | 0.1016 | 0.0103 |  |  |  |  |  | 3 | 0.872  (0.776-0.979) | 0.525-1.000 | 0.1016 | 0.0103 |
|  | ML | 3 | 0.657  (0.620-0.696) |  | 0.0481 | 0.0023 | 3 | 0.723  (0.671-0.779) |  | 0.0623 | 0.0039 | 6 | 0.689  (0.650-0.731) | 0.565-0.841 | 0.0712 | 0.0051 |
|  | DL |  |  |  |  |  |  |  |  |  |  |  |  |  |  |  |
|  | Overall | 6 | 0.757  (0.661-0.867) | 0.474-1.000 | 0.1686 | 0.0284 | 3 | 0.723  (0.672-0.779) | 0.528-0.990 | 0.0623 | 0.0039 | 9 | 0.746  (0.680-0.817) | 0.532-1.000 | 0.1392 | 0.0194 |
| MRI features |  |  |  |  |  |  |  |  |  |  |  |  |  |  |  |  |
|  | LR |  |  |  |  |  |  |  |  |  |  |  |  |  |  |  |
|  | ML | 2 | 0.785  (0.658-0.937) |  | 0.1269 | 0.0161 | 1 | 0.750  (0.673-0.836) |  |  |  | 3 | 0.775  (0.691-0.869) | 0.478-1.000 | 0.0959 | 0.0092 |
|  | DL | 2 | 0.833  (0.746-0.929) |  | 0.0691 | 0.0048 | 1 | 0.820  (0.747-0.900) |  |  |  | 3 | 0.835  (0.781-0.893) | 0.657-1.000 | 0.0443 | 0.0020 |
|  | Overall | 4 | 0.804  (0.731-0.885) | 0.576-1.000 | 0.0929 | 0.0086 | 2 | 0.788  (0.722-0.859) |  | 0.0362 | 0.0013 | 6 | 0.799  (0.746-0.857) | 0.640-0.998 | 0.0787 | 0.0062 |
| X-Ray features |  |  |  |  |  |  |  |  |  |  |  |  |  |  |  |  |
|  | LR |  |  |  |  |  |  |  |  |  |  |  |  |  |  |  |
|  | ML |  |  |  |  |  |  |  |  |  |  |  |  |  |  |  |
|  | DL | 1 | 0.613  (0.579-0.649) |  |  |  | 1 | 0.770  (0.733-0.809) |  |  |  | 2 | 0.687  (0.550-0.859) |  | 0.1589 | 0.0253 |
|  | Overall | 1 | 0.613  (0.579-0.649) |  |  |  | 1 | 0.770  (0.733-0.809) |  |  |  | 2 | 0.687  (0.550-0.859) |  | 0.1589 | 0.0253 |
| MRI + Clinical features |  |  |  |  |  |  |  |  |  |  |  |  |  |  |  |  |
|  | LR |  |  |  |  |  | 2 | 0.709  (0.653-0.769) |  | 0.0273 | 0.0007 | 2 | 0.709  (0.653-0.769) |  | 0.0273 | 0.0007 |
|  | ML | 1 | 0.763  (0.673-0.865) |  |  |  | 2 | 0.837  (0.774-0.906) |  | 0 | 0 | 3 | 0.815  (0.763-0.872) | 0.704-0.944 | 0 | 0 |
|  | DL | 3 | 0.878  (0.862-0.894) |  | 0 | 0 | 1 | 0.850  (0.782-0.924) |  |  |  | 4 | 0.877  (0.861-0.893) | 0.851-0.903 | 0 | 0 |
|  | Overall | 4 | 0.875  (0.859-0.892) | 0.848-0.903 | 0.0026 | 0.0001 | 5 | 0.786  (0.720-0.858) | 0.603-1.000 | 0.0847 | 0.0072 | 9 | 0.820  (0.773-0.869) | 0.676-0.994 | 0.0779 | 0.0061 |
| X-Ray + Clinical features |  |  |  |  |  |  |  |  |  |  |  |  |  |  |  |  |
|  | LR | 1 | 0.742  (0.719-0.765) |  |  |  |  |  |  |  |  | 1 | 0.742  (0.719-0.765) |  |  |  |
|  | ML | 3 | 0.706  (0.649-0.767) |  | 0.0732 | 0.0054 | 4 | 0.864  (0.846-0.883) |  | 0.0086 | 0.0001 | 7 | 0.792  (0.725-0.866) | 0.581-1.000 | 0.1184 | 0.0140 |
|  | DL |  |  |  |  |  | 1 | 0.807  (0.771-0.842) |  |  |  | 1 | 0.807  (0.771-0.842) |  |  |  |
|  | Overall | 4 | 0.714  (0.670-0.762) | 0.568-0.899 | 0.0643 | 0.0041 | 5 | 0.854  (0.827-0.882) | 0.778-0.938 | 0.0296 | 0.0009 | 9 | 0.788  (0.735-0.845) | 0.610-1.000 | 0.1049 | 0.0110 |
| X-Ray + MRI + Clinical features |  |  |  |  |  |  |  |  |  |  |  |  |  |  |  |  |
|  | LR |  |  |  |  |  | 4 | 0.617  (0.576-0.660) |  | 0 | 0 | 4 | 0.617  (0.576-0.660) | 0.552-0.689 | 0 | 0 |
|  | ML | 1 | 0.730  (0.723-0.737) |  |  |  | 5 | 0.785  (0.688-0.896) |  | 0.1460 | 0.0213 | 6 | 0.776  (0.695-0.865) | 0.535-1.000 | 0.1331 | 0.0177 |
|  | DL |  |  |  |  |  |  |  |  |  |  |  |  |  |  |  |
|  | Overall | 1 | 0.730  (0.723-0.737) |  |  |  | 9 | 0.709  (0.634-0.793) | 0.476-1.000 | 0.1631 | 0.0266 | 10 | 0.712  (0.645-0.787) | 0.495-1.000 | 0.1524 | 0.0232 |

Note: n: number of models; PI: prediction interval; LR: logistic regression; ML:machine learning; DL: deep learning.

**Table S6** Results of meta-analysis of sensitivity and specificity of ML for predicting other progression of KOA

| Modeling variables | Model | Cross-validation | | | Independent validation | | | | Overall | | |
| --- | --- | --- | --- | --- | --- | --- | --- | --- | --- | --- | --- |
|  |  | n | SE(95%CI) | SP(95%CI) | n | SE(95%CI) | SP(95%CI) | | n | SE(95%CI) | SP(95%CI) |
| Clinical features |  |  |  |  |  |  | |  |  |  |  |
|  | LR | 2 | 0.78-0.92 | 0.78-0.89 |  |  | |  | 2 | 0.78-0.92 | 0.78-0.89 |
|  | ML | 2 | 0.65-0.68 | 0.61-0.79 | 3 | 0.32-0.67 | | 0.64-0.90 | 5 | 0.57(0.45-0.69) | 0.79(0.67-0.87) |
|  | DL |  |  |  |  |  | |  |  |  |  |
|  | Overall | 4 | 0.77(0.63-0.87) | 0.79(0.68-0.87) | 3 | 0.32-0.67 | | 0.64-0.90 | 7 | 0.67(0.51-0.80) | 0.80(0.72-0.87) |
| MRI features |  |  |  |  |  |  | |  |  |  |  |
|  | LR |  |  |  |  |  | |  |  |  |  |
|  | ML | 2 | 0.67-0.80 | 0.78-0.79 | 1 | 0.80 | | 0.63 | 3 | 0.67-0.80 | 0.63-0.79 |
|  | DL | 2 | 0.67-0.85 | 0.72-0.79 | 1 | 0.62 | | 0.83 | 3 | 0.62-0.85 | 0.72-0.83 |
|  | Overall | 4 | 0.75(0.66-0.82) | 0.78(0.76-0.80) | 2 | 0.62-0.80 | | 0.63-0.83 | 6 | 0.74(0.66-0.81) | 0.77(0.73-0.80) |
| X-Ray features |  |  |  |  |  |  | |  |  |  |  |
|  | LR |  |  |  |  |  | |  |  |  |  |
|  | ML |  |  |  |  |  | |  |  |  |  |
|  | DL |  |  |  | 1 | 0.77 | | 0.71 | 1 | 0.77 | 0.71 |
|  | Overall |  |  |  | 1 | 0.77 | | 0.71 | 1 | 0.77 | 0.71 |
| MRI + Clinical features |  |  |  |  |  |  | |  |  |  |  |
|  | LR |  |  |  | 2 | 0.65-0.76 | | 0.66-0.69 | 2 | 0.65-0.76 | 0.66-0.69 |
|  | ML | 1 | 0.66 | 0.76 | 2 | 0.75-0.78 | | 0.78-0.81 | 3 | 0.66-0.78 | 0.76-0.81 |
|  | DL | 3 | 0.78-0.83 | 0.73-0.85 | 1 | 0.85 | | 0.69 | 4 | 0.82(0.77-0.86) | 0.78(0.71-0.83) |
|  | Overall | 4 | 0.78(0.70-0.84) | 0.79(0.73-0.83) | 5 | 0.76(0.69-0.81) | | 0.73(0.67-0.78) | 9 | 0.77(0.72-0.81) | 0.76(0.71-0.80) |
| X-Ray + Clinical features |  |  |  |  |  |  | |  |  |  |  |
|  | LR | 1 | 0.66 | 0.80 |  |  | |  | 1 | 0.66 | 0.80 |
|  | ML | 3 | 0.60-0.71 | 0.70-0.86 | 2 | 0.72-0.89 | | 0.82-0.83 | 5 | 0.68(0.64-0.72) | 0.80(0.75-0.84) |
|  | DL |  |  |  | 1 | 0.72 | | 0.81 | 1 | 0.72 | 0.81 |
|  | Overall | 4 | 0.66(0.62-0.69) | 0.79(0.72-0.84) | 3 | 0.72-0.89 | | 0.81-0.83 | 7 | 0.68(0.65-0.71) | 0.80(0.76-0.83) |
| X-Ray + MRI + Clinical features |  |  |  |  |  |  | |  |  |  |  |
|  | LR |  |  |  | 4 | 0.58(0.41-0.72) | | 0.64(0.50-0.76) | 4 | 0.58(0.41-0.72) | 0.64(0.50-0.76) |
|  | ML | 1 | 0.68 | 0.79 | 5 | 0.71(0.53-0.84) | | 0.75(0.61-0.85) | 6 | 0.70(0.56-0.81) | 0.76(0.65-0.85) |
|  | DL |  |  |  |  |  | |  |  |  |  |
|  | Overall | 1 | 0.68 | 0.79 | 9 | 0.65(0.52-0.76) | | 0.70(0.59-0.79) | 10 | 0.66(0.54-0.75) | 0.71(0.61-0.80) |

Note: n: number of models; LR: logistic regression; ML: machine learning; DL: deep learning; SE: sensitivity; SP: specificity.
